# Amyloid-β Plaques in Clinical Alzheimer’s Disease Brain Incorporate Stable Isotope Tracer *In Vivo* and Exhibit Nanoscale Heterogeneity

**DOI:** 10.3389/fneur.2018.00169

**Published:** 2018-03-22

**Authors:** Norelle C. Wildburger, Frank Gyngard, Christelle Guillermier, Bruce W. Patterson, Donald Elbert, Kwasi G. Mawuenyega, Theresa Schneider, Karen Green, Robyn Roth, Robert E. Schmidt, Nigel J. Cairns, Tammie L. S. Benzinger, Matthew L. Steinhauser, Randall J. Bateman

**Affiliations:** ^1^Department of Neurology, Washington University School of Medicine, St. Louis, MO, United States; ^2^Department of Physics, Washington University in St. Louis, St. Louis, MO, United States; ^3^NanoImaging Center, Division of Genetics, Brigham and Women’s Hospital, Cambridge, MA, United States; ^4^Brigham and Women’s Hospital, Boston, MA, United States; ^5^Harvard Medical School, Boston, MA, United States; ^6^Department of Medicine, Washington University School of Medicine, St. Louis, MO, United States; ^7^Department of Neurology, The University of Texas at Austin Dell Medical School, Austin, TX, United States; ^8^Department of Pathology and Immunology, Washington University School of Medicine, St. Louis, MO, United States; ^9^Department of Cell Biology and Physiology, Washington University School of Medicine, St. Louis, MO, United States; ^10^Washington University Center for Cellular Imaging, Washington University School of Medicine, St. Louis, MO, United States; ^11^Knight Alzheimer’s Disease Research Center, Department of Neurology, Washington University School of Medicine, St Louis, MO, United States; ^12^Hope Center for Neurological Disorders, Department of Neurology, Washington University School of Medicine, St. Louis, MO, United States; ^13^Department of Radiology, Washington University School of Medicine, St. Louis, MO, United States; ^14^Department of Neurological Surgery, Washington University School of Medicine, St. Louis, MO, United States

**Keywords:** Alzheimer’s disease, Aβ plaques, plaque dynamics, stable isotope tracer, SILK–SIMS

## Abstract

Alzheimer’s disease (AD) is a neurodegenerative disorder with clinical manifestations of progressive memory decline and loss of executive function and language. AD affects an estimated 5.3 million Americans alone and is the most common form of age-related dementia with a rapidly growing prevalence among the aging population—those 65 years of age or older. AD is characterized by accumulation of aggregated amyloid-beta (Aβ) in the brain, which leads to one of the pathological hallmarks of AD—Aβ plaques. As a result, Aβ plaques have been extensively studied after being first described over a century ago. Advances in brain imaging and quantitative measures of Aβ in biological fluids have yielded insight into the time course of plaque development decades before and after AD symptom onset. However, despite the fundamental role of Aβ plaques in AD, *in vivo* measures of individual plaque growth, growth distribution, and dynamics are still lacking. To address this question, we combined stable isotope labeling kinetics (SILK) and nanoscale secondary ion mass spectrometry (NanoSIMS) imaging in an approach termed SILK–SIMS to resolve plaque dynamics in three human AD brains. In human AD brain, plaques exhibit incorporation of a stable isotope tracer. Tracer enrichment was highly variable between plaques and the spatial distribution asymmetric with both quiescent and active nanometer sub-regions of tracer incorporation. These data reveal that Aβ plaques are dynamic structures with deposition rates over days indicating a highly active process. Here, we report the first, direct quantitative measures of *in vivo* deposition into plaques in human AD brain. Our SILK–SIMS studies will provide invaluable information on plaque dynamics in the normal and diseased brain and offer many new avenues for investigation into pathological mechanisms of the disease, with implications for therapeutic development.

## Introduction

Alzheimer’s disease (AD) is a devastating neurodegenerative disorder characterized by progressive cognitive and functional decline and is the most common form of age-related dementia (1, 2). The incidence of AD-related death and disability is rapidly growing in persons 65 years of age or older whose risk of developing the disease doubles every 5 years (3); currently, an estimated 5.3 million Americans are affected (4). Amyloid-beta (Aβ), a 38–43 amino acid peptide derived from the proteolytic cleavage of the amyloid precursor protein, is implicated as a pathogenic species in AD (5–10). Abnormal accumulation and aggregation of Aβ in the cerebral extracellular space result in one of the pathological hallmarks of AD—amyloid plaques. As a result, the onset and rate of amyloidosis has been intensively studied over the past several decades. Although older individuals may have extensive Aβ deposition without clinical signs of dementia (11–14), there is a strong association of amyloid plaques with AD dementia. Longitudinal measures of amyloid pathology have shown increases in amyloid deposition 15–20 years prior to dementia onset (15) in early onset, familial AD and predict future cognitive decline (16–20).

As amyloid pathology increases *in vivo*, a concomitant decline in cerebrospinal fluid (CSF) Aβ42 occurs (21). More recent studies in the production and clearance of Aβ in CSF (22) demonstrate that the clearance of Aβ42 is decreased in AD. Intriguingly, with the onset of amyloidosis, only the Aβ42 proteoform (23, 24) demonstrated faster turnover kinetics attributed to rapid deposition into plaques—for ~50% of all Aβ42 produced (22). Further, there was a positive correlation between increased Aβ42 turnover kinetics and rate of amyloidosis measured by PET (22). In plasma, the altered Aβ42 turnover kinetics and lower Aβ42/40 ratio with amyloidosis is also present albeit to a lesser extent (25). These data suggest that alterations in Aβ concentrations and kinetics are strongly associated with amyloidosis reflecting deposition of Aβ into plaques.

Longitudinal PET studies demonstrate that amyloid plaque pathology increases slowly and plateaus with the clinical presentation of dementia (15, 20, 26–29). The slow accumulation of amyloid tracer binding over decades before clinical presentation of dementia has been suggestive of stability in plaque accumulation. The plateau of amyloid tracer binding may indicate that amyloid pathology reaches dynamic equilibrium or quiescence globally at the clinical stage of dementia. Similarly, several animal model studies of plaques have demonstrated that plaques grow, typically at earlier ages (30–32), to a stable size (32–34), but that plaques may be in equilibrium with their environment if continual deposition is assumed (33).

However, despite the relevance of amyloid plaques in AD, measures of *in vivo* growth and distribution of human plaques are lacking due to analytical challenges and limitations. Previous human *in vivo* studies (22, 25, 35) relied on CSF and plasma, which are indirect measures of the brain compartment and do not directly measure plaques. PET imaging of human brain is an average measure of amyloid pathology over ~1 cm, which does not measure individual amyloid plaques, and amyloid tracers bind only a subset of aggregated Aβ (36). Interpretation of PET results is limited because amyloid binding agents measure binding sites and not necessarily the true amount of amyloid plaques. Fluorescence-based assessments using multi-photon imaging of over-expression models, which do not fully replicate the human disease (37–39), encounter limitations resulting from specificity of dyes, decreased sensitivity due to tissue autofluorescence, and the inability to distinguish new versus previously existing Aβ or other proteins found deposited into plaques (40). As individual plaque dynamics have not been directly measured in humans, we therefore sought to measure the *in vivo* incorporation of protein into amyloid plaques to resolve whether plaques are stable or dynamic structures in the human AD brain.

Stable isotope labeling kinetics (SILK) was developed to determine protein production and clearance by administering a stable isotope labeled precursor *in vivo* and sampling during and after labeling (41). The development of SILK allowed quantitation of protein turnover in humans, providing a direct readout of changes underlying pathophysiology of disease and pharmacodynamics of therapeutics (22, 24, 42). However, SILK performed from CSF or plasma does not convey localization of labeled biomolecules in tissue. Nanoscale secondary ion mass spectrometry (NanoSIMS) allows both imaging and measurement of stable isotopes at high spatial resolution (50–100 nm or <1 μm^3^) (43, 44). While NanoSIMS has been applied to the study of AD (45, 46), it has not been used in combination with SILK for the measurement of *in vivo* protein translation in cells or deposition into plaques in normal or diseased brain. Here, we coupled SILK to NanoSIMS in a method termed SILK–SIMS to directly measure and image the distribution and rate of protein deposition (a proxy for growth taking into account area) into individual plaques at the nanometer level (44, 47–49). Our aim was to measure the stability or growth of individual amyloid plaques in AD animal models and in the human brain.

Initial SILK–SIMS experiments in a mouse model of AD demonstrated *in vivo* detection of the stable isotope tracer ^13^C_6_-leucine in native Aβ plaques. Further, plaques incorporated more of the tracer via protein deposition compared to protein translation in the surrounding brain parenchyma, but less than neurons. In human AD brain, we found ^13^C enrichment in multiple diffuse and dense-core plaques in both the frontal lobe and precuneus. ^13^C enrichment was highly variable and asymmetric between plaques with both quiescent and active nanometer sub-regions of tracer incorporation. These data reveal that human Aβ plaques are dynamic structures and suggest that they have growth or turnover rates that can be directly quantified in AD brain. Our results provide the first, direct measures of *in vivo* plaque dynamics in human AD brain. We predict that SILK–SIMS will provide a precise understanding of protein deposition into amyloid plaques in human AD brain and other CNS disturbances characterized by protein aggregation.

## Materials and Methods

### Cell Culture

B-cell hybridoma line (produced in-house by Holtzman Lab, Washington University) was grown for 5 days in leucine-free media that was supplemented with either ^12^C_6_-leucine or ^13^C_6_-leucine at 26 mg/L and mixed at the appropriate percentage of heavy isotope-containing media with 2% FBS. Cells were harvested and spun at 1,000 rpm for 5 min at room temperature (RT). Cell pellets were resuspended in 4°C Ringers wash solution for 5 min, spun, and then fixed with 4% paraformaldehyde in 100 mM NaCl, 30 mM HEPES, 2 mM CaCl_2_, pH 7.2 (NaHCa) for 2 h. This was followed by three rinses of NaHCa at RT and overnight incubation at 4°C in NaHCa. Centrifugation was used throughout the following steps in order to re-concentrate the cells to a pellet. The following morning, pellets were placed into ddH_2_O and then infiltrated with LR White Embedding Media (Catalog #14383, EMS, Hatfield, PA) using the manufacturer’s published protocol with minor modification. Partial dehydration was accomplished by using 20% EtOH 15 min, 40% EtOH 15 min, 50% EtOH 15 min, 70% EtOH 15 min, 85% EtOH 10 min, followed by 1 h in a 2:1 LR White to 85% EtOH. Sections of LR White embedded samples were cut on a Leica UC7 Ultramicrotome using a diamond knife. 200 nm and 400 nm sections were picked up with a perfect loop, placed on top of a polished silicon (Si) wafer (Catalog #534, University Wafer Inc., South Boston, MA, USA), and let air dry on a 35°C hot plate.

### Animals

All animal procedures were conducted in accordance with the animal protocol approved by the Washington University Animal Studies Committee and are consistent with the National Institutes of Health (NIH) guidelines for the care and use of animals. Two male double transgenic mice expressing chimeric mouse/human amyloid precursor protein (Mo/HuAPP695swe) and a mutant human presenilin 1 (PS1-dE9) both directed to CNS neurons (stock 34832-JAX) (50) were kindly provided by Dr. Timothy Miller. Animals were given leucine-free chow (Catalog #1831936, Test Diet, St. Louis, MO, USA) with 5 mg/mL ^12^C_6_-leucine added to 2% sucrose-containing drinking water to control leucine intake for a one-week acclimation period. After the 1-week acclimation period, animals were given 5 mg/mL ^13^C_6_- or ^12^C_6_-leucine orally *via* 2% sucrose drinking water, averaging 36 mL of H_2_O/week. Animals were 4 months old at the time of labeling (pre-plaque pathology) and 6.5 months old at the end of labeling (onset of plaque pathology). Following the end of the labeling paradigm, animals were anesthetized with 65 mg/kg pentobarbital sodium and sacrificed by decapitation. Brains were removed and placed in 10% neutral-buffer formalin (Catalog #15740-01, EMS, Hatfield, PA, USA). Pieces of mouse brain were washed into NaHCa and incubated overnight at 4°C. The following morning, samples were stained with 1% osmium/NaHCa for 1 h, washed four times over 1 h, and then *en bloc* stained with 1% uranyl acetate/H_2_O for 1 h in the dark. Samples were rinsed with three exchanges of water, 10 min each, and then processed for LR White embedding as described above with the addition of being gold-coated once on the Si wafer. Serial adjacent sections were placed on glass microscope slides for toluidine blue staining for light microscopy.

### APP/PS1 Plasma Leucine

Mouse whole blood was spun at 1,000 × *g* for 10 min, and the plasma (supernatant) was removed. Plasma proteins were precipitated with ice-cold acetone followed by de-lipidation with hexane, and the aqueous fraction was dried *in vacuo* (51). 1:1N-Methyl-N-tert-butyldimethylsilyltrifluoroacetamide/acetonitrile was added, and samples were incubated at 70°C for 30 min. Duplicate 1 µL injections were made into an Agilent 5973 MSD mass spectrometer using a 30 µm × 0.25 mm DB-5MS column (Agilent Technologies). Electron impact ionization and selected ion monitoring were used to measure endogenous unlabeled leucine at *m/z* 200 (molecular ion minus C-1 as CO_2_-tert-butyldimethylsilyl; CO2-tBDMS), and ^13^C_6_-leucine (tracer) was measured at *m/z* 205 as an m + 5 ion. The tracer-to-tracee ratio (TTR) was taken as the m + 5/m + 0 peak area ratio of the biological sample minus the m + 5/m + 0 ratio of a natural abundance leucine sample. The molar fraction of labeled leucine was calculated as: MFL = TTR/(1 + TTR).

### Human Tissue

Clinically and neuropathologically well-characterized human brain tissue samples were obtained from the Charles F. and Joanne Knight Alzheimer’s Disease Research Center (Knight ADRC), Washington University School of Medicine, Saint Louis, Missouri. At the time of death, written informed consent in accordance with the Declaration of Helsinki was obtained from the next-of-kin in accordance with the protocol approved by the Washington University Human Studies Committee and the General Clinical Research Center Advisory Committee. Cognitive status at expiration was determined using a validated retrospective post-mortem interview with an informant to establish the clinical dementia rating (CDR) (52). We used frozen tissue from the frontal lobe (Brodmann areas 8/9) of Pt2 with mild AD dementia (CDR 1, age at death = 88 years; post-mortem interval = 15 h; post-labeling interval (delta) = 8 days; Table 1). The right cerebral hemisphere was coronally sliced at 1 cm intervals and snap frozen by contact with pre-cooled Teflon^®^-coated aluminum plates, and temperature equilibrated by immersion into liquid nitrogen vapor in a cryo-vessel. Following freezing, tissues were placed in Ziploc^®^ storage bags and stored in freezer at −80°C. Participants 2 and 3 were given the stable isotope tracer ^13^C_6_-leucine as part of previous SILK studies (22, 35). These AD participants had made previous arrangements for brain donation to Washington University.

**Table 1:** Patient demographics for SILK-SIMS imaging of Aβ plaques.

Participant	Amyloid status PET-PiB	CDR^a^	AD dementia	MMSE^a^	Gender	Age	Δ Between labeling and DOE (days)
1	N/A	N/A	N/A	N/A	M	59	N/A^b^
2	pos	1	Mild	22	M	88	1,150 and 8^c^
3	pos	1	Mild	11	M	88	1,648

*Clinical autopsy provided a diagnosis of Alzheimer’s disease (AD) for all participants*.

*CDR, clinical dementia rating; MMSE, mini mental state exam; DOE, date of expiration*.

*^a^CDR and MMSE as most recent*.

*^b^This participant was not part of the SILK studies*.

*^c^This participant was in two SILK studies and thus has two time lapses*.

Formalin-fixed tissue from the frontal lobe, including the cortical ribbon, of Pt1 (negative control, no labeling), Pt2 (delta = 8 days), and Pt3 (delta = 4.5 years) were post-fixed in 2% osmium tetroxide in 0.1 M sodium cacodylate buffer for 1 h, *en bloc* stained with 3% aqueous uranyl acetate for 1 h, dehydrated in graded ethanols, and embeded in PolyBed 812 catalog #08792-1 (Polysciences, Hatfield, PA, USA). Blocks were polymerized at 80°C for 72 h. Tissue blocks were sectioned using a diamond ultrathin section knife on a Reichert Ultra-Cut E ultramicrotome at 300–500 nm. Sections were transferred to a single polished Si wafer for SILK–SIMS analysis. Serial adjacent sections were placed on glass microscope slides for toluidine blue staining for light microscopy. The precuneus of Pt2 was prepared and embedded in LR White as described above for animal tissue along with samples from the 10-week labeled APP/PS1 mouse (positive control) and Pt1 (negative control). Serial adjacent sections were placed on glass microscope slides for toluidine blue staining for light microscopy.

### Light Microscopy

Toluidine blue stained sections were imaged with a Hamamatsu NanoZoomer 2.0-HT System. Imaging was done to guide feature identification and location for electron microscopy.

### Electron Microscopy

Images of the tissue and reference points were taken with a field emission scanning electron microscope (FE-SEM; Quanta^TM^ 3D FEG, FEI, Hillsboro, OR, USA) in order to document plaque locations and provide an absolute coordinate system for the tissue. In-house coordinate transformation software was used to translate tissue regions-of-interest (ROIs) and reference points found in the FE-SEM to the NanoSIMS instrument stage coordinate plane for relocation of the same ROIs. Additional sections were cut at 70–90 nm for transmission electron microscopy (JEOL JEM-1400Plus) to image selected plaques to define ultrastructure. Anti-Aβ antibody 82E1 (1:50; Aβ N-terminal epitope (1-16); IBL-America, Minneapolis, MN, USA) was used with goat anti-mouse secondary antibody conjugated to 10 nm gold particles (1:15).

### Magnetic Resonance Imaging (MRI) and PET-PiB Imaging

Participants were labeled with the radiotracer N-methyl-2-(4-methylaminophenyl)-6-hydroxybenzothiazole (Pittsburgh compound B, PiB) for human brain PET imaging of amyloid deposition. PiB was prepared as previously described (53). PET and MRI were performed as previously described (54).

### NanoSIMS

Data were acquired on either a Cameca NanoSIMS 50 at Washington University (cells and human tissue) or NanoSIMS 50 L at Brigham and Women’s Hospital (mouse tissue). Images of the B-cell hybridoma used to calculate the ^13^C_6_-leucine standard curve were acquired with a 50 µm primary beam raster for 15 min at 1 ms/px and 65.5 s/plane (dwell time) for a total of 10 planes (i.e., cycles) per mass (256 × 256 px). APP/PS1 mouse brain tissue was acquired at a 17–60 µm raster for 11–87 min at 2 ms/px and 131 s/plane (dwell time) for a total of 5–40 cycles (i.e., planes) per mass (256 × 256 px). For all APP/PS1 measurements, the non-labeled cells (0%) were measured prior to the SILK–SIMS data acquisition. The unlabeled cells and the natural abundance of carbon-13 (55) were used for quantitation of normalized ratios for mouse plaques and neuron. Plaque ROI outlines were drawn based on plaque morphology and areas around the plaque defined such they do not overlap or intersect with the predefined plaque ROIs.

The human tissue was analyzed in three sets. In the first set (frontal lobe), Pt1 (negative control) and Pt2 frontal lobe were embedded in PolyBed 812 and pre-sputtered using the D1-1 aperture for 10 min followed by data acquisition at a raster size between 35 and 50 µm raster with a D1-2 aperture. Data acquisition was 4 h at 5 ms/px and 327.68 s/plane (dwell time) for a total of 40 planes (i.e., cycles) per mass (256 × 256 px). In the second set (precuneus), Pt2 precuneus samples (embedded in LR White; Figures S9 and S11 in Supplementary Material) were pre-sputtered at 30 µm raster with a D1-1 aperture for 10 min followed by data acquisition at 25 µm raster with the D1-2 aperture. Data acquisition was 2.5 h at 5 ms/px and 327.68 s/plane (dwell time) for a total of 25 planes (i.e., cycles) per mass (256 × 256 px). Pt1, also embedded in LR White, was used as the negative control in this experimental set. Finally, unlabeled AD tissue (Pt1; in Supplementary Material) embedded in PolyBed812 was acquired with a 45 µm raster for 11.6 h at 4 ms/px and 1,048.576 s/plane (dwell time) for a total of 40 planes (i.e., cycles) per mass (512 × 512 px). Pt3’s (in Supplementary Material) brain tissue was acquired with a 55 µm raster for 18 h at 5 ms/px and 1,310 s/plane (dwell time) for a total of 40 planes (*i.e*., cycles) per mass (512 × 512 px). For all measurements, the non-labeled material (Pt1) was measured prior to the SILK–SIMS data acquisition. In this way, the negative control data were acquired first before SILK data acquisition, and the electron multipliers were no longer adjusted after negative control data were acquired.

### NanoSIMS Data Analysis

Each analysis was performed in 24-h blocks with measurements on a *SiC* standard of known isotopic composition to assess instrument stability followed by measurements on an unlabeled control prior to SILK–SIMS data acquisition. Raw image data were imported into the L’Image image processing software (Larry Nittler, Carnegie Institution of Washington) to produce quantitative ratio images of heavy and light isotopes and determine where isotopic anomalies were located. Images were automatically segmented into 10 × 10 pixel ROIs using L’image, and the heavy/light isotopic ratios were calculated from the summed ion counts within each ROI. The fractional uncertainty, ƒ, of each ROI ratio heavy/light isotope ratios in each region-of-interest (ROI) was calculated in Excel as the sum in quadrature of the standard deviation of the average ratios measured for non-labeled material, σ_Std_, and the Poisson errors, σ_ROI_, of the ROI itself, as given by the equation

(1) $$f=\sqrt{{\left(\frac{\sigma_{\text{Std}}}{R_{\text{Std}}}\right)}^{2}+{\left(\frac{\sigma_{\text{ROI}}}{R_{\text{ROI}}}\right)}^{2}}$$

where *R*_Std_ is the average ratio of repeated measurements on unlabeled tissue, and *R*_ROI_ is the ratio calculated from summing the counts of every pixel contained within the individually defined ROI. This uncertainty represents the entire experimental precision and accuracy, including: counting statistics, matrix effects, systematic error, instrumental tuning, and differences between standards and samples. From this uncertainty, the amount, significance, and location of heavy isotopic labeling can be quantitatively determined. Those ROIs with heavy/light isotope ratios greater than or equal to the μ + 2σ value of the unlabeled sample were analyzed by a one-sample *t*-test. Two SDs from the mean (2σ) was chosen to ensure high confidence in isotope enrichment (i.e., 95%) prior to the one-sample *t*-test; by analogy in the field of Physics, a minimum 5σ effect is required for statistical confidence. Each *t*-value of the resulting *t*-tests was corrected for multiple testing with a FDR of 0.01 using the Benjamini-Hochberg method (56). Those ROIs which remained significant after correcting for multiple comparisons remain outlined in the images.

### Aβ Extraction

1 g of frontal lobe tissue was homogenized in ice-cold 1X PBS with 0.05% CHAPS and centrifuged at 17,000 × *g* for 30 min at 4°C as previously described (57). The supernatant was spun for 1 h at 100,000 × *g* at 4°C, and the resulting pellet was solubilized in 5 M guanidine overnight at 4°C with rotation. Next, samples were spun for 20 min at 17,000 × *g* at 4°C, and the supernatant was diluted 1:10 in 1X PBS in BSA-block tubes as previously described (57). Samples were immunoprecipitated (IP) with 50 μL of HJ5.1 anti-Aβ antibody [mid-domain epitope (17–28)] coupled Dynabeads (Catalog #14311D, Invitrogen, Carlsbad, CA, USA) made following the manufacturer’s instructions. After overnight incubation at 4°C, IPs were eluted in neat formic acid and dried *in vacuo*. Samples were resuspended in 50 mM TEABC (Catalog #17902, Sigma, St. Louis, MO, USA), spiked with ^15^N-Aβ internal standard, and digested overnight with 0.25 ng/µL Lys-N (Catalog #100965-1, Seikagaku Biobusiness Corp., Tokyo, Japan) at 4°C.

### Mass Spectrometry

Samples were resuspended in 1% FA/10% ACN (*v/v*) with 20 nM BSA digest (Catalog #1863078, Pierce, Rockford, IL, USA). Samples were analyzed in triplicate by nanoLC-MS/MS on an LTQ-Orbitrap Fusion (Thermo Fisher Scientific) in positive ion mode. Separations were performed using an online NanoAcquity UPLC (Waters, Milford, MA, USA) using an ACQUITY UPLC HSS T3 (360 µm OD × 75 µm ID) column packed with 10 cm C_18_ (1.8 µm, 100 Å, Waters) at 300 nL/min and heated to 65°C. Mobile phases were 0.1% FA in water (A) and 0.1% FA in ACN (B). Samples were eluted from the column with the gradient starting at 12% B, which was ramped to 32% B over 10 min and further increased to 90% B over 5 min and held for 1 min, before re-equilibration to 12% B over 2 min. Total run time, including column equilibration, sample loading, and analysis, was 30 min. The mass spectrometer was operated in targeted MS2 mode. MS2 spectra were acquired in the Orbitrap (30,000 at *m/z* 400) in centroid mode using XCalibur, version 4.0 (Thermo Fisher Scientific). Ion injection times for the targeted MS2 scans for labeled and unlabeled samples, respectively (in ms) were: Aβ mid-domain (54, 1080), Aβ40 (54, 540), and Aβ42 (54, 1080). The Orbitrap automatic gain control targets were set to 5 × 10^5^ for all proteoforms except Aβ42, which was set to 1 × 10^6^. The targeted precursor ions were sequentially isolated in the quadrupole and fragmented in the Orbitrap using HCD (isolation width 1.6 Da, normalized collision energy 25%, activation *Q* 0.250, and activation time 10 ms). The general mass spectrometric conditions were as follows: spray voltage 2.2 kV, 60% S-lens, and ion transfer tube temperature 275°C.

### Mass Spectrometry Data Analysis

Data (.raw files) were imported into a Skyline template containing the Lys-N C-terminal peptides of Aβ38, 40, 42, and the mid-domain. Retention time alignment was based on the ^15^N internal standard. The sum of all transitions (*b* ions monitored for each parent peptide) for unlabeled and ^13^C_6_-leucine labeled Aβ peptides were exported from Skyline to Excel. The ratios of labeled/unlabeled of each replicate (triplicate injections) for Pt2 sample were taken followed by isotopic background subtraction of the mean ratio of an unlabeled participant to give the TTR minus background for each replicate injection. Next, the background subtracted TTRs were used to calculate the mean and SD (i.e., the mean of the area ratios) of enrichment for each Aβ peptide.

### Data Availability Statement

NanoSIMS image files (.im) and L’Image saved session (.sav) files are available upon request from corresponding authors. Excel data analysis files for human NanoSIMS images can be found in the Supplementary Material.

## Results

### Quantification of ^13^C and *In Vivo* Labeling

**Figure 1:**
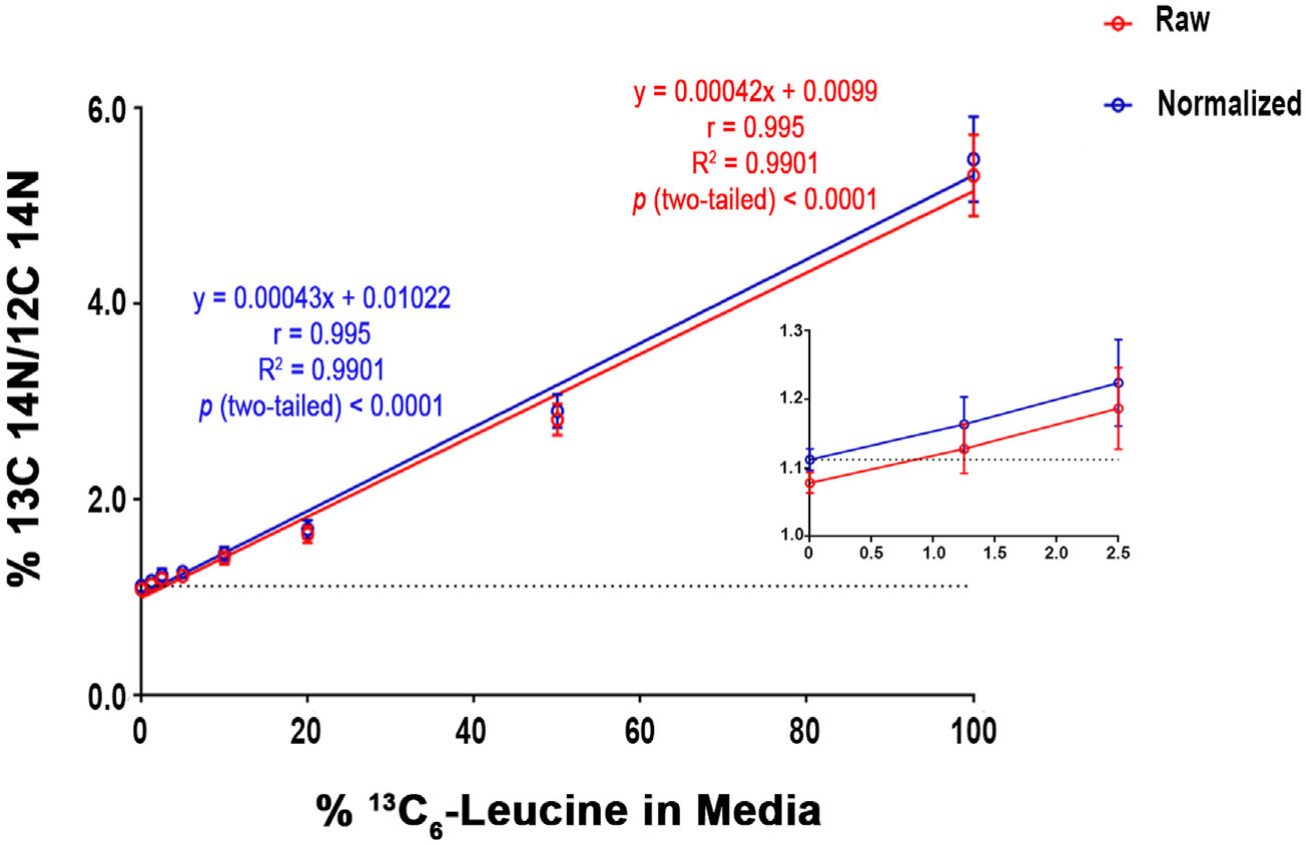
SILK-SIMS dose response curve for ^13^C_6_-leucine enrichment. B-cell hybridoma cells were labeled with increasing percentages of ^13^C_6_-leucine. The standard curve demonstrates a monotonic relationship between detected ^13^C^14^N/^12^C^14^N and the percent ^13^C_6_-leucine enrichment in cell media. Red line represents the mean of ^13^C^14^N/^12^C^14^N ± SD of the regions-of-interest outlined in (Figure S3 in Supplementary Material). The blue line represents the normalized mean ^13^C^14^N/^12^C^14^N ± SD. Each standard curve point was normalized (Equation 4 in Supplementary Material) to the ratio of natural abundance ^13^C and unlabeled cells (0%). The normalized SD was calculated as the sum in quadrature of the SD of the average ratios measured for non-labeled cells (0%) and the Poisson errors of the feature itself (Equation 5 in Supplementary Material). Note the expected intercept at ~1.1%. *Inset*: enlarged view of the three lowest points on the dose-response curve. Enrichments as low as 1.25% were detectible above background. Dashed horizontal line represents natural abundance of ^13^C (1.1%).

**Figure 2:**
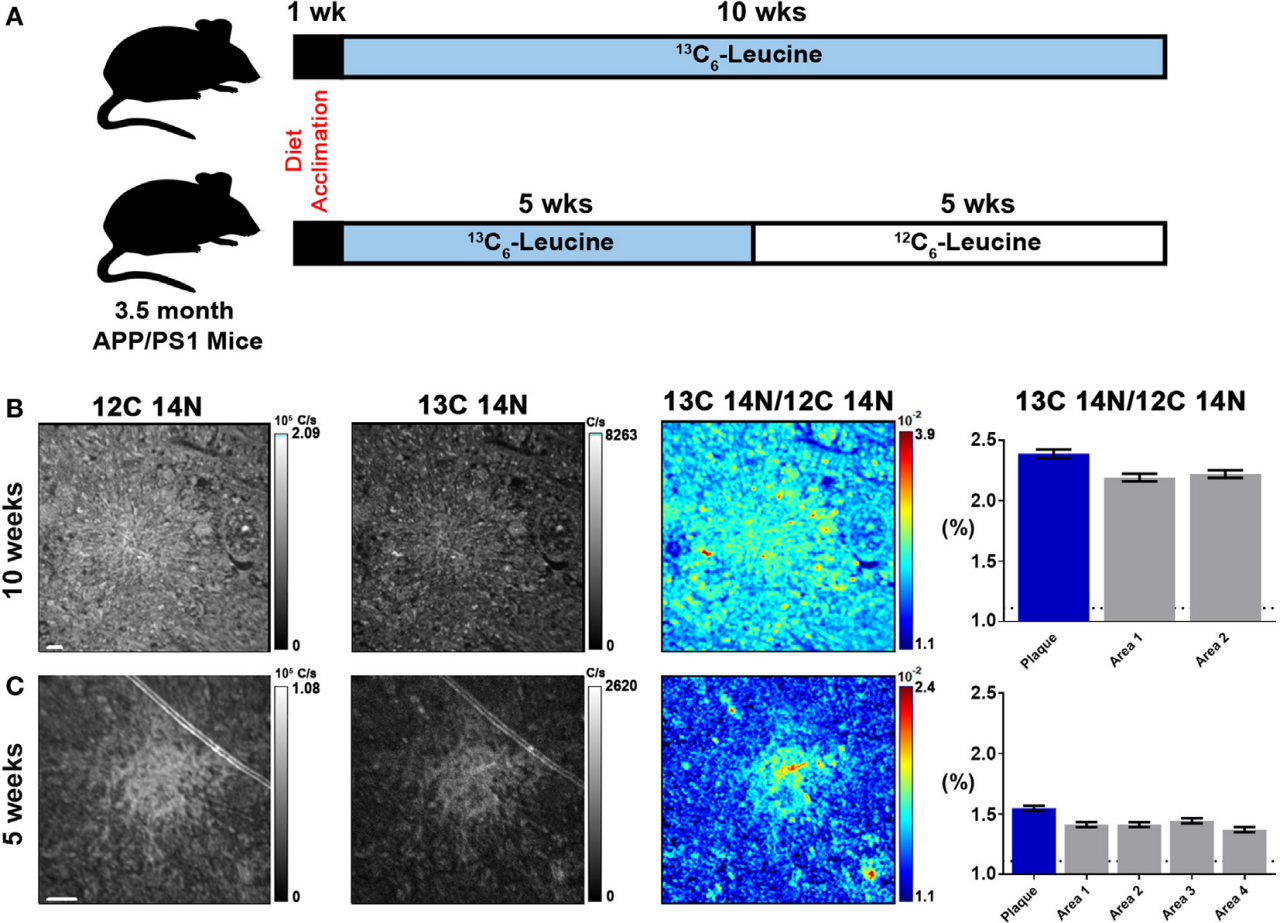
Stable isotope incorporation within plaques of APP/PS1 mouse model of Alzheimer’s disease. **(A)** Labeling scheme to determine the feasibility of detecting *in vivo* incorporation of stable isotope ^13^C in native Aβ plaques. **(B,C)** From left to right, ^12^C^14^N ion map, ^13^C^14^N ion map, ^13^C^14^N/^12^C^14^N ion map, and normalized ^13^C^14^N/^12^C^14^N ratios of 10-week and 5-week labeled APP/PS1 mice, respectively (35 × 35 μm images; see, Figure S4 in Supplementary Material, for region-of-interest (ROI) outlines based on plaque morphology and areas around the plaque defined such they do not overlap with plaque ROIs). Error bars indicate ± SD in quadrature of the SD of the average ratios measured for non-labeled cells (0%) and the Poisson errors of the feature itself (Equation 5 in Supplementary Material). Dashed horizontal line represents natural abundance of ^13^C (1.1%). *Scale bar*, 2 µm.

To quantify carbon isotopes in biological material, we measured carbon as negative ions of ^12^C, ^13^C, ^12^C^14^N, and ^13^C^14^N. Using a B-cell hybridoma grown in leucine-free media supplemented with increasing percentages of ^13^C_6_-leucine, we characterized the signal response for ^13^C enrichment. Figure 1 demonstrates the linearity of response of SILK–SIMS measurements to increasing isotopic enrichment of ^13^C_6_-leucine in culture measured as the ratio of ^13^C^14^N/^12^C^14^N, which produced improved image quality and more accurate quantification (Figure S1–3 in Supplementary Material). To determine whether ^13^C_6_-leucine enrichment could be detected *in vivo* in native Aβ plaques, two individual APP/PS1 mice (3.5 months of age) were administered ^13^C_6_-leucine tracer for 10 and 5 weeks (Figure 2A). The mouse labeled for 10 weeks (Figure 2B) reached a ^13^C^14^N/^12^C^14^N ratio of 2.4% ± 0.03% in plaque (expected unlabeled at 1.1% due to ^13^C natural abundance) compared to brain parenchyma (2.2% ± 0.03%) in both Area 1 and Area 2 (Figure 2B; Figure S4 in Supplementary Material). A plaque from the APP/PS1 mouse labeled for 5 weeks, followed by a 5-week washout, remained enriched at 1.6% ± 0.02% relative to natural abundance (Figure 2C), and compared to adjacent areas (1.4% ± 0.02%). Despite being labeled for half the amount of time with an additional 5 weeks of tracer washout, the plaque enrichment was approximately two-thirds of that measured in the 10-week labeled animal (1.6% ± 0.02% vs. 2.4% ± 0.03%). The enrichment of plasma leucine measurements taken at the time of collection was fourfold lower (72 vs. 16% isotopically labeled leucine in the 10-week vs. 5-week labeled mouse, Table 1). A neuron incorporated substantially more tracer than the surrounding parenchyma (neuron = 3.3% ± 0.05% vs. area 1 = 2.4% ± 0.03% vs. area 2 = 2.4% ± 0.03%) and the plaque (Figure S5 in Supplementary Material) in the 10-week labeled mouse. These results demonstrate that ^13^C_6_-leucine incorporation into Aβ plaques and other features could be measured and is localized to protein deposition (i.e., plaques) and protein translation (i.e., cells).

### *In Vivo* Labeling and Quantification of Human AD Plaques

**Figure 3:**
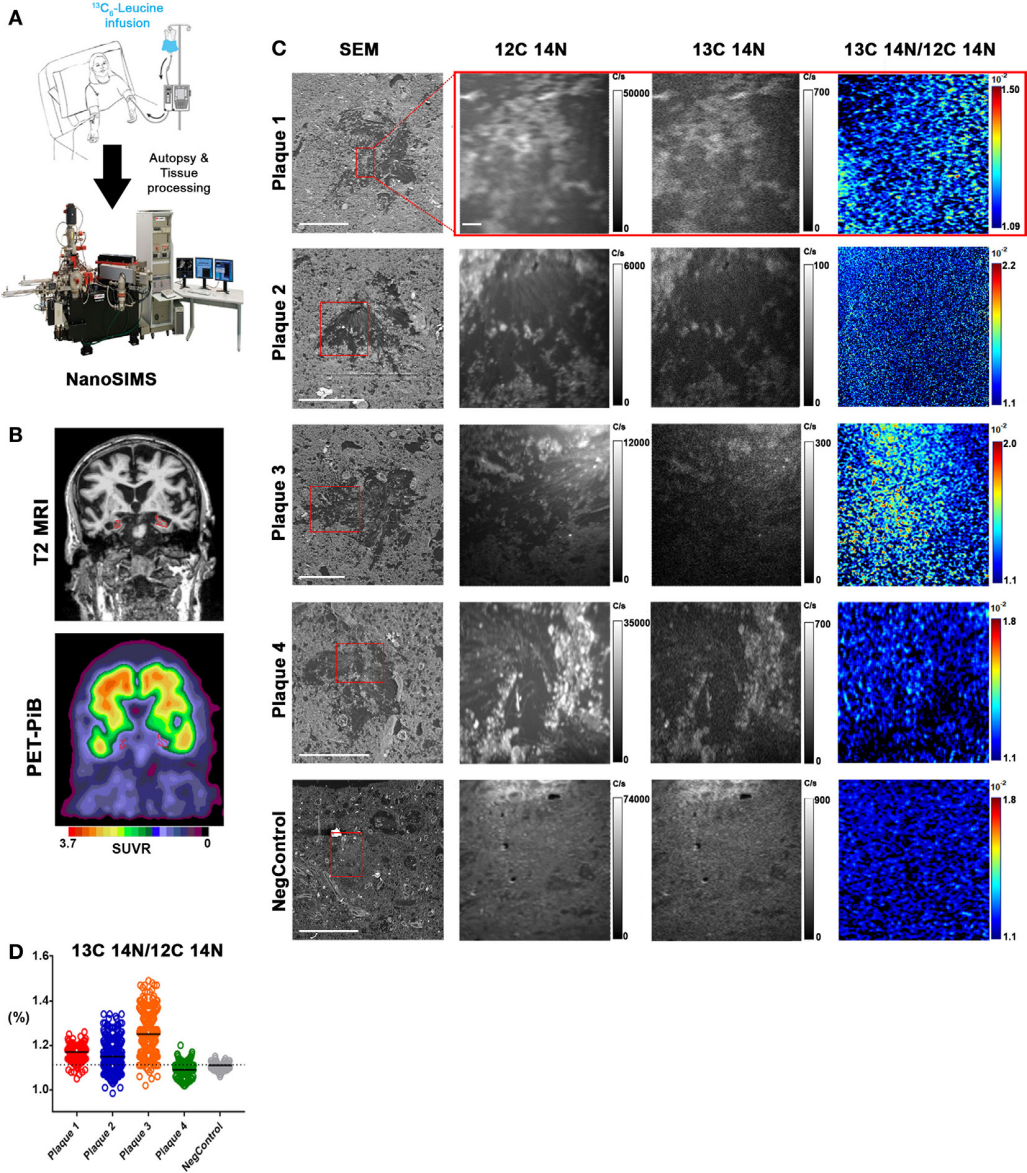
*In vivo* stable isotope incorporation of ^13^C within plaques in the frontal lobe of clinically and pathologically confirmed Alzheimer’s disease brain. **(A)** Primary question: are amyloid-β plaques dynamic structures able to incorporate the stable isotope tracer ^13^C_6_-leucine and can this be directly measured in the human brain? **(B)** Traditional imaging of SILK Pt2 by T2-weighted magnetic resonance imaging (MRI) (*upper*) and by PET-PiB (*lower*) in a coronal view. Hippocampal atrophy (*red*) in the MRI image is apparent along with prominent PiB binding in the PET image. SUVR, standardized uptake value ratio. **(C)** Four diffuse plaques in the frontal lobe of SILK Pt2 and a dense core plaque from the frontal lobe of non-SILK Pt1 (Table 2). From left to right, scanning electron microscope (SEM) image of the plaque (red box defines area imaged by SILK–SIMS), ^12^C^14^N ion map, ^13^C^14^N ion map, and ^13^C^14^N/^12^C^14^N ion map. **(D)** Summary scatter plot of all plaque region-of-interest normalized ratios shown below. Horizontal bars represent plaque ^13^C^14^N/^12^C^14^N medians, and dashed horizontal line represents natural abundance of ^13^C (1.1%). *Scale bar* for all SEM images, 40 µm. *Scale bar* for all SILK–SIMS images, 5 µm. SEM magnification and SILK–SIMS image raster, respectively: Plaque 1: 636X and 25 × 25 μm; Plaque 2: 1,024 and 35 × 35 μm; Plaque 3: 628X and 50 × 50 μm; Plaque 4: 628X and 45 × 45 μm; NegControl: 600X and 45 × 45 μm.

**Figure 4:**
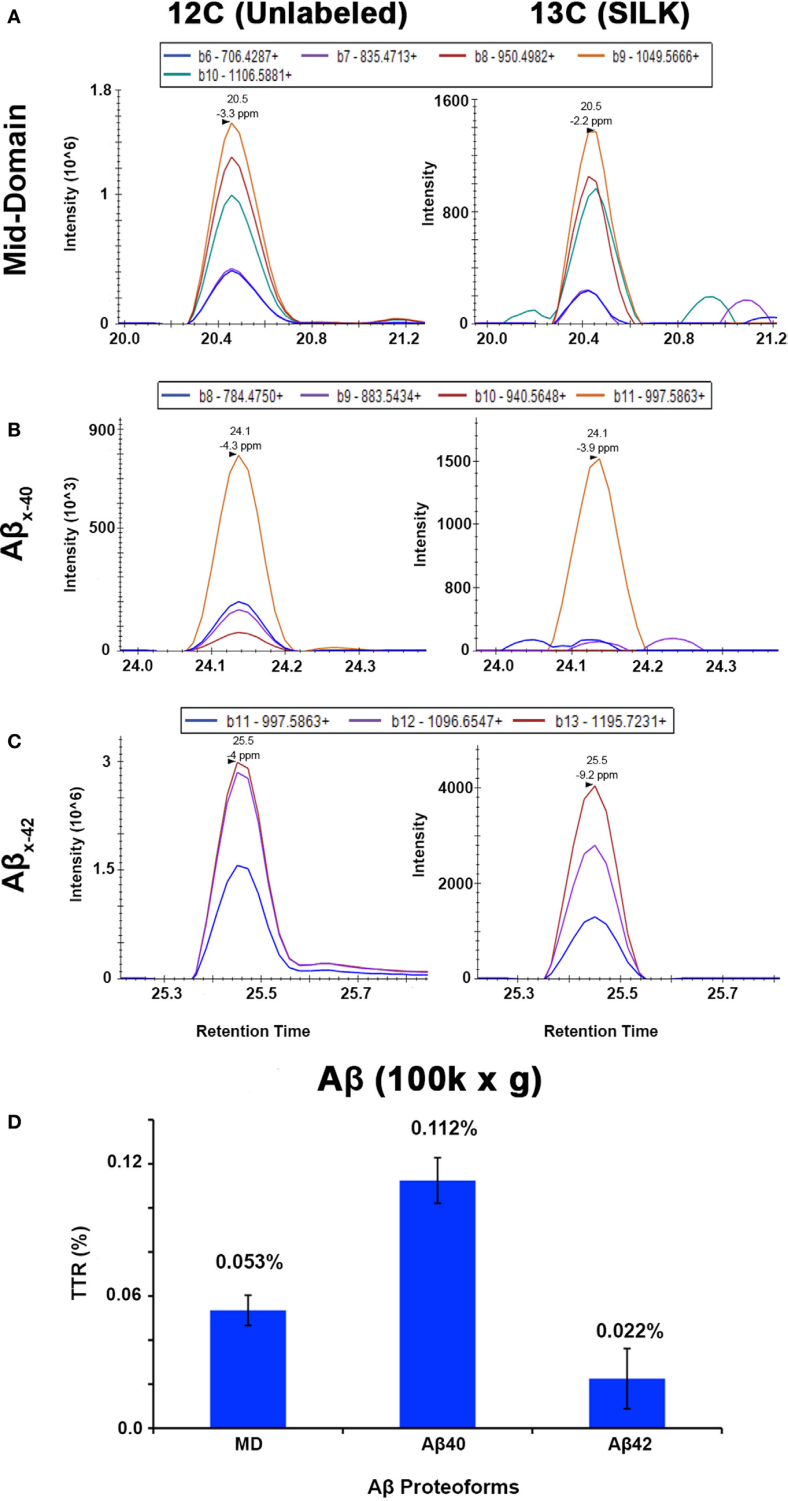
Targeted nLC-MS/MS of Aβ proteoforms from an insoluble fraction of SILK Pt2. Extracted ion chromatograms (XIC) are shown for **(A)** Aβ mid-domain, **(B)** Aβ_x-40_, and **(C)** Aβ_x-42_ in Pt2 (8 day delta between labeling and expiration) of unlabeled Aβ and ^13^C_6_-leucine labeled (*SILK*), respectively. **(D)** The average of each tracer-to-tracee ratio (TTR) from triplicate injections using targeted nLC-MS/MS after background subtraction from an unlabeled participant sample. The relative quantitation of Aβ mid-domain, Aβ_x-40_, and Aβ_x-42_ provides additional evidence of ^13^C_6_-leucine present in the brain even after supposed label washout. Error bars represent ± SD.

The APP/PS1 mouse model (50) is an over-expression model of APP mutations typically found in early onset, familial disease, which accounts for <1% of AD cases (58) and does not replicate all aspects of the human disease or human amyloid-plaques (37–39). Further, despite intensive study of plaque dynamics in animal models (30–34), little is known about individual plaque dynamics in human AD brain. The demonstrated feasibility of measuring ^13^C_6_-leucine incorporation in native Aβ plaques from mouse brain (Figure 2) prompted us to investigate post-mortem tissue from previous SILK study participants (22, 35). Plaques from unlabeled AD participant tissue (Table 2; Figure S6 in Supplementary Material) were similar to the natural abundance of ^13^C as expected. In a previous SILK participant (Pt3) (22), who had a post-labeling interval of 4.5 years (Figure S7 in Supplementary Material), we were also unable to detect ^13^C enrichment in the examined areas. In contrast, post-mortem tissue from Pt2, who was labeled (35) and passed away 8 days later due to an unrelated cause, had significant ^13^C enrichment in amyloid plaques (Figure 3). Four diffuse plaques in the frontal lobe displayed puncta of ^13^C labeling intercalated in the periphery and fibrillar interior (Figure 3C). Notably, not all plaques demonstrated equivalent amounts of ^13^C incorporation or the same number of enriched regions (max ^13^C = 1.26, 1.34, 1.49, and 1.20%, for plaques 1–4, respectively). Among plaques with an equivalent number of enriched regions (e.g., plaques 2 and 3) ^13^C enrichment varied, suggesting differing rates of ^13^C incorporation among “dynamic” plaques (Figures 3C,D). Additionally, the number of areas with significant enrichment drastically differed, indicating that plaque incorporation of ^13^C is highly asymmetric and can be constrained to specific regions or sub-regions; in the case of plaque 4, it is confined to a 3.0 µm^2^ area (Figures 3C,D; Figure S8 in Supplementary Material). To confirm this result was not restricted to a single brain region, we examined plaques in the precuneus (Figure S9 in Supplementary Material). Of the two dense-core plaques imaged by SILK–SIMS and verified by immunoelectron microscopy (Figure S10 in Supplementary Material), only plaque 5 had ^13^C enrichment within the plaque, the peri-plaque region (Figures S9A–F in Supplementary Material), and up to 50 µm away (Figure S11 in Supplementary Material). In contrast, plaque 6 had a low number of enriched regions, which appear part of the surrounding brain parenchyma (Figures S9G–L in Supplementary Material) rather than the plaque itself, suggesting a quiescent plaque. To provide orthogonal validation of the presence of ^13^C_6_-leucine Aβ proteoforms (23) in Pt2 brain, we used targeted nLC-MS/MS. In Aβ from insoluble aggregates (57), we quantified ^13^C_6_-leucine labeled Aβx-40, 42, and mid-domain peptides, which were enriched at relative abundances of 0.112, 0.022, and 0.053% above natural abundance, respectively, while Aβx-38 was not detected as expected (Figure 4).

**Table 2:** Plasma leucine in labeled APP/PS1 mice.

	^12^C_6_-Leucine peak area	^13^C_6_-Leucine peak area	^13^C/^12^C	TTR	Mol fraction labeled	Average Mol fraction labeled
10 week	4,579	11,793	258%	258%	72%	72%
	4,601	11,845	257%	257%	72%	
5 week	14,438	2,878	20%	20%	16%	16%
	14,256	2,724	19%	19%	16%	

*Each sample was injected twice with excellent reproducibility*.

*TTR, tracer-to-tracee ratio*.

## Discussion

In the current study, we utilized brain tissue from participants enrolled in previous SILK studies (22, 35) for NanoSIMS imaging. During the *in vivo* stable isotope labeling procedure (41) newly synthesized proteins are labeled simultaneously. As a result, many different features (e.g., plaques, parenchyma, and neurons) and kinetic processes (e.g., protein translation, transport, deposition, and clearance) in the brain can be imaged directly without the reliance of analytical intermediaries or modifications such as fluorescence. In the previous SILK studies, ^13^C_6_-leucine was used as the tracer (41, 59). However, in SILK-SIMS, ^13^C is isotopically diluted, decreasing sensitivity, due to the abundance of carbon in mammalian tissues and the carbon-based embedding media. As a result, we were only able to successfully detect ^13^C enrichment in one participant who had a post-labeling interval of eight days. Yet, to the best of our knowledge, this represents the first report of imaging of individual human amyloid plaques *in vivo* dynamics at the nanometer scale not appreciable by previous methods.

### Human Aβ Plaques Are Dynamic Structures

The incorporation of ^13^C_6_-leucine into plaques (i.e., protein deposition) in a participant with clinical stage AD dementia suggests that plaques can be dynamic with activity over days even in later stages of the disease. Notably, we were able to demonstrate dynamic activity in both diffuse and dense-core plaques in contrast to previous animal model studies (30–34), which were restricted to imaging dense-core plaques due to the use of fluorescent plaque binding reagents Thioflavin S and Methoxy-04. The “*activity*” of individual plaques can be highly variable as demonstrated by varying isotope enrichment, suggesting differing rates of protein incorporation even among individual plaques in the same brain region. A single plaque could simultaneously demonstrate nanometer scale regions of dynamic incorporation of the tracer as well as quiescence—highly asymmetric tracer incorporation.

Plaque asymmetry was previously suggested (33) in a mouse model of AD but was attributed to asymmetrical clearance. The quiescence regions and sub-regions of individual plaques could be interpreted as either asymmetric clearance or differential nanometer scale deposition. However, not all plaques incorporated the tracer indicating a lack of protein deposition during the same timeframe. Together, these results demonstrate that plaques can be at both dynamic equilibrium and quiescence within a single participant even with dementia, at both the individual plaque level and plaque nanometer sub-region level, despite PET studies suggesting no net change (20, 26–29).

### Relationship to Previous Studies and Limitations

Previous visualizations of plaque formation examined by multi-photon imaging have arrived at differing conclusions (30–34). This may be due to a potential significant impact on plaque response with surgical intervention and the use of over-expression models *versus* direct human pathophysiology. Multi-photon imaging has the advantages of visualization of individual plaques over time in the same animal non-destructively albeit at lower resolution limit compared to SILK–SIMS. In contrast, SILK–SIMS is a destructive technique where the isotope profiles are obtained at the expense of the molecular identities of the newly synthesized and deposited biomolecules. This is particularly relevant as plaques are heterogeneous structures containing other proteins, cellular debris, and lipids (40), which may contribute to the ^13^C signal seen in the SILK-SIMS images. As Aβ is the main constituent of plaques, we validated ^13^C enrichment by nLC-MS/MS from the same sample in an orthogonal manner. This orthogonal measure can be applied to other proteins of interest (e.g., alpha-synuclein, tau, and prion protein), or MALDI imaging (60, 61) may be used in parallel to obtain molecular identities of plaque constituents *in situ*, though at micrometer resolution.

## Conclusion and Outlook

The application of SILK–SIMS to future studies will allow estimates of the rate and distribution of plaque deposition at the nanometer level across human AD cohorts. The results will provide a better understanding of the rate of AD pathophysiology and may have a significant impact on the development of therapeutic strategies; for example, drugs targeting amyloid plaque growth and resorption through a variety of mechanisms. However, SILK–SIMS, unlike PET imaging, is currently limited to post-mortem tissue. Biopsies are an alternative but are invasive and only feasible with surgery to relieve other conditions such as normal pressure hydrocephalus. Nonetheless, SILK–SIMS may also be applied to measuring other proteinaceous deposits such as Lewy bodies (alpha-synuclein), neurofibrillary tangles (tau), and prion protein, which are characteristic of Parkinson’s disease, frontotemporal dementia and AD, and Creutzfeldt–Jakob disease, respectively. In general, the SILK–SIMS approach can be used to visualize incorporation of new biomolecules at the nanometer scale to measure growth and turnover to better understand physiology and pathophysiology of many disease states, neurological and non-neurological.

## Ethics Statement

All animal procedures were conducted in accordance with the animal protocol approved by the Washington University Animal Studies Committee and are consistent with the National Institutes of Health (NIH) guidelines for the care and use of animals. Clinically and neuropathologically well-characterized human brain tissue samples were obtained from the Charles F. and Joanne Knight Alzheimer’s Disease Research Center (Knight ADRC), Washington University School of Medicine, Saint Louis, Missouri. At the time of death, written informed consent in accordance with the Declaration of Helsinki was obtained from the next-of-kin in accordance with the protocol approved by the Washington University Human Studies Committee and the General Clinical Research Center Advisory Committee.

## Author Contributions

NW, DE, FG, and RB designed the experiments. NW, FG, and CG operated the NanoSIMS instrument for SILK–SIMS data acquisition. NW performed all animal experiments and biochemical fractionation of SILK AD tissue. BP contributed APP/PS1 and human plasma leucine measurements, data analysis, and critical discussions regarding relevance of NanoSIMS to Aβ peptide SILK studies. KM assisted with mass spectrometry data acquisition and analysis. TS performed sample digestion and preparation for mass spectrometry analysis. KG and RR performed histology and EM sample preparation. RS provided unlabeled tissue, supervised the EM preparation, and confirmed plaque pathology. NC provided SILK tissue and advised about plaque location in tissue and sample processing. TB provided PET-PiB and MRI images and analysis. MS and RB supervised analyses and provided critical feedback at all junctures. NW made the figures. NW, FG, and RB wrote the manuscript. All authors approved the manuscript.

## Conflict of Interest Statement

RB and Dr. Holtzman (Chair of the Department of Neurology) and Washington University in St. Louis have equity ownership interest in C2N Diagnostics and may receive royalty income based on technology licensed by Washington University to C2N Diagnostics. In addition, RB and Dr. Holtzman receive income from C2N Diagnostics for serving on the Scientific Advisory Board. Washington University, with RB and Dr. Holtzman as co-inventors, has also submitted the U.S. non-provisional patent application “Methods for measuring the metabolism of CNS derived biomolecules *in vivo*,” serial #12/267,974. Washington University, with NW, RB, FG, and DE, has also submitted the U.S. provisional patent application “Methods and systems for measuring, detecting, and determining the location of a biomolecule or drug,” #62523811.
