# Supplementary material for: Amyloid-β Plaques in Clinical Alzheimer’s Disease Brain Incorporate Stable Isotope Tracer In Vivo and Exhibit Nanoscale Heterogeneity
Source: Front Neurol. 2018 Mar 22;9:169. doi: 10.3389/fneur.2018.00169 (PMC5874304; doi:10.3389/fneur.2018.00169)
Supplement: Supplementary file 1 [file presentation_1.PDF]

Supplementary Material

Amyloid-β plaques in clinical Alzheimer's disease brain incorporate stable isotope tracer *in vivo* and exhibit nanoscale heterogeneity

Authors: Norelle C. Wildburger*, Frank Gyngard, Christelle Guillermier, Bruce W. Patterson, Donald Elbert, Kwasi G. Mawuenyega, Theresa Schneider, Karen Green, Robyn Roth, Robert E. Schmidt, Nigel J. Cairns, Tammie L.S. Benzinger, Matthew L. Steinhauser, and Randall J. Bateman*

*** Correspondence:** Corresponding Author: n.wildburger@wustl.edu and batemanr@wustl.edu

1 Supplementary Figures
2 Formulas
3 Supplementary Datasets
4 Supplementary References

1.1 Supplementary Figures

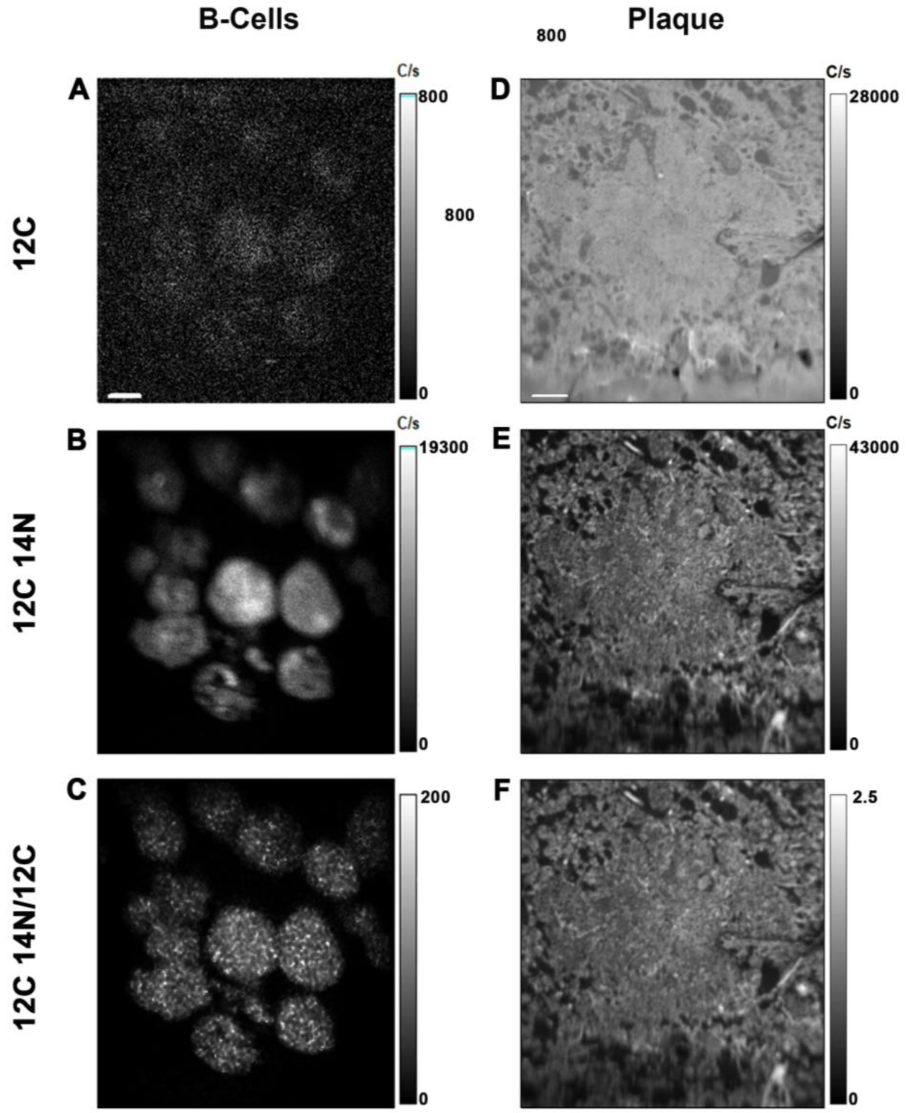

Supplementary Figure 1: Image quality of carbon imaged as monoisotopes (^{12}C and ^{13}C) and polyatomic isotopes (^{12}C^{14}N and ^{13}C^{14}N). (**A-C**) 50×50 µm image of 0% ^{13}C$_6$-leucine labeled cells imaged by SILK-SIMS with electron multipliers set to detect ^{12}C and ^{12}C^{14}N ions, respectively. (**D-F**) 45×45 µm image of an unlabeled human AD plaque (Pt1) imaged by SILK-SIMS with electron multipliers set to detect ^{12}C and ^{12}C^{14}N ions, respectively. (**C, F**) The ratio of ^{12}C^{14}N/^{12}C is used deduce the nitrogen contribution of the polyatomic isotope image in 0% labeled cells and unlabeled human AD plaque, respectively. Apparent from panels (**B)** and (**E**) is the improved image quality, morphology, and counts per second (C/s) when carbon is imaged as a polyatomic isotope (*i.e.*, cyanide ion) compared to carbon monoisotopes, panels (**A**) and (**D**). The This is likely due to *i*) the higher ionization potential of cyanide ions compared to carbon as evidenced by the signal intensity (*i.e.*, C/s), *ii*) the contribution of nitrogen, which is abundant in biological materials. The nitrogen content of biological materials with CN$^-$ molecules most abundant in proteins (18%) compared to RNA and DNA (1.1% and 0.25%) (1,2) and *iii*) reduced carbon contribution from the embedding media (1). Note, that in the case of embedded B-cells, nitrogen localization (panel **C**) is restricted to the cells rather than the embedding matrix (background; *i.e.*, black area). This is in contrast to the ^{12}C ion map, which show carbon signals coming from the background. *Scale bar*, 5 µm.

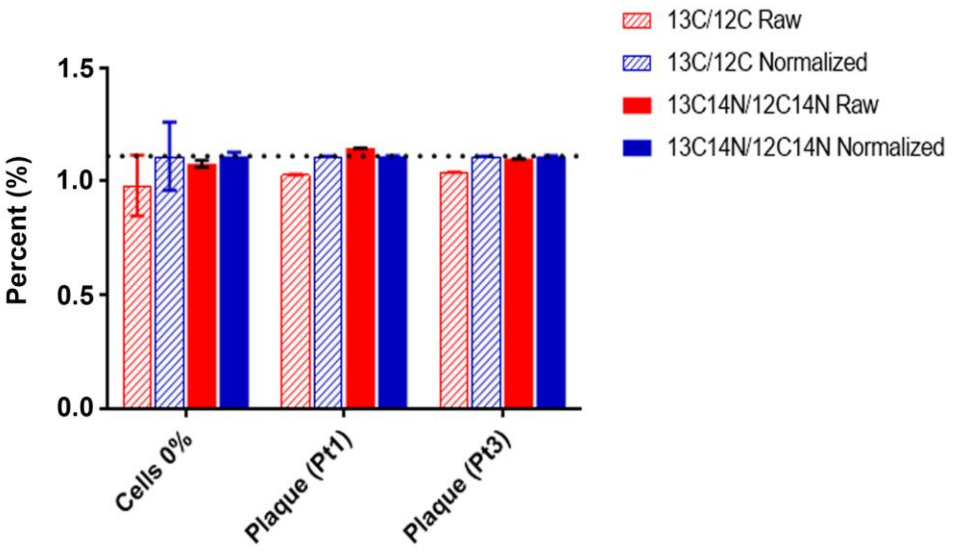

Supplementary Figure 2: Quantitative improvement in image analysis using polyatomic carbon isotopes *versus* monoisotopes. Raw and normalized values of $^{13}C/^{12}C$ and $^{13}C^{14}N/^{12}C^{14}N$ ratios for 0% $^{13}C_6$-leucine labeled cells, unlabeled human AD plaque (Pt1), and SILK Pt3 with a delta of 4.5 yrs between labeling and expiration. Raw values represent the mean ± S.D. of all $^{13}C/^{12}C$ and $^{13}C^{14}N/^{12}C^{14}N$ ratios across the entire image over all cycles of that image. Normalized values are the raw ratios normalized to the natural abundance of ^{13}C (1.1%) and their respective standard deviations were calculated as the sum in quadrature of the standard deviation of the average ratios measured for non-labeled material and the Poisson errors of the feature itself. Quantitatively, CN^- molecules provide improved accuracy and precision compared to C^- alone. Dashed horizontal line represents natural abundance of ^{13}C.

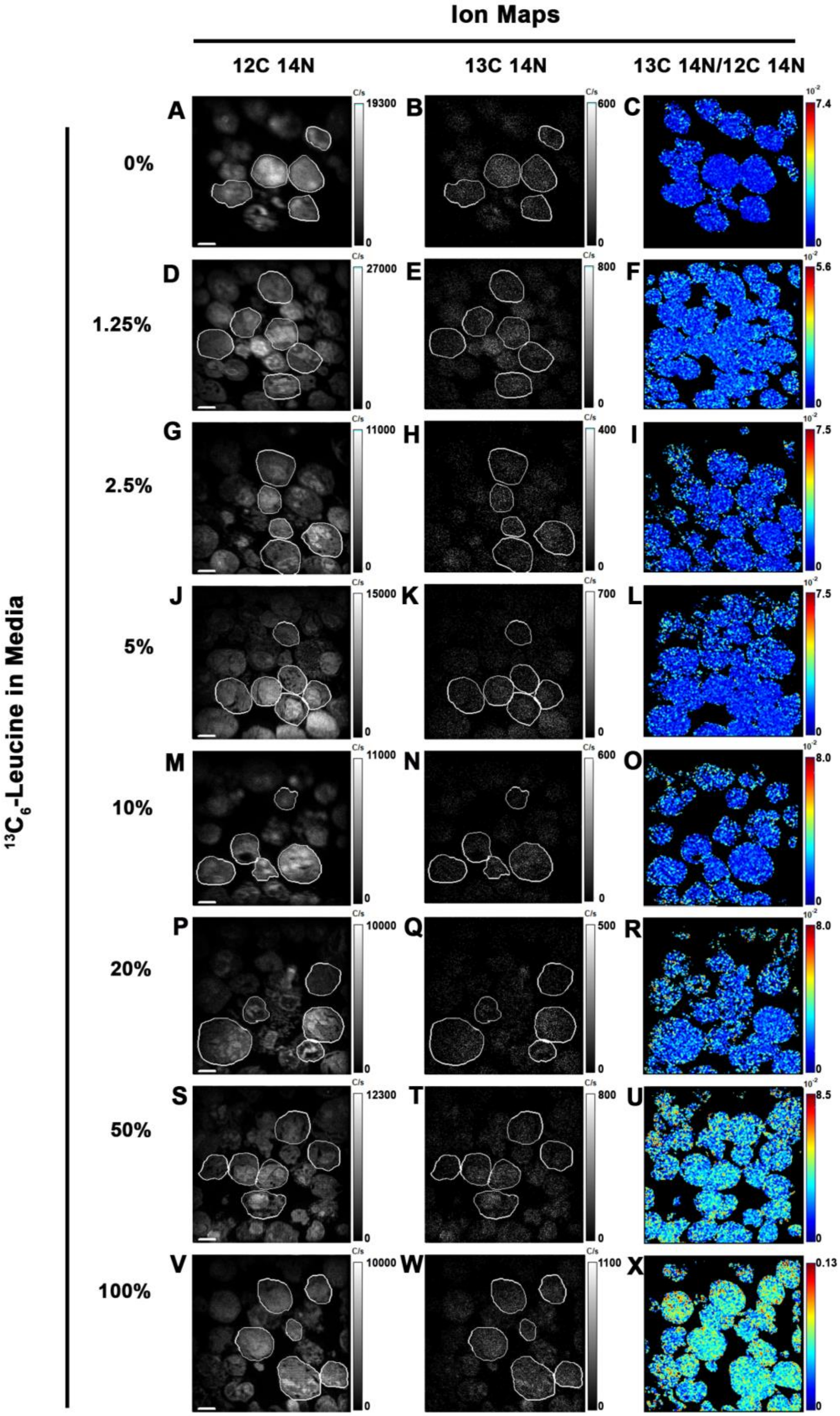

Supplementary Figure 3: Quantitative imaging of increasing ^{13}C enrichment in cell-based standard curve. *Left to right*, $^{12}C^{14}N$, $^{13}C^{14}N$, and $^{13}C^{14}N/^{12}C^{14}N$ ion maps of 50×50 μm images of B-cell hybridoma

given increasing percentages of $^{13}C_6$-leucine in the cell media to generate the standard curve seen in **Figure 1**. *Scale bar*, 5 μm.

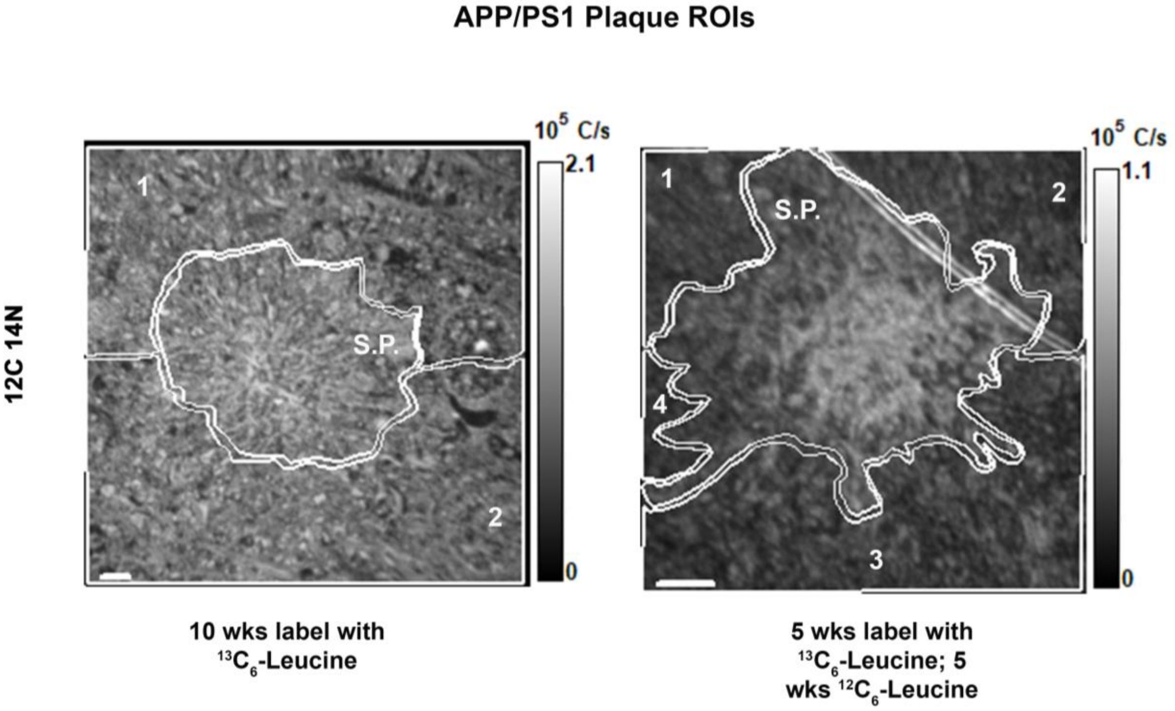

Supplementary Figure 4: ROIs used for isotope enrichment quantitation in APP/PS1 mice. Morphological outline (*white*) of the ROIs used define senile plaques (S.P.) and areas (#1-2 and #1-4) in APP/PS1 mice labeled for 10 weeks and 5 weeks, respectively, for quantitation in **Figure 2**. *Scale bar*, 2 μm

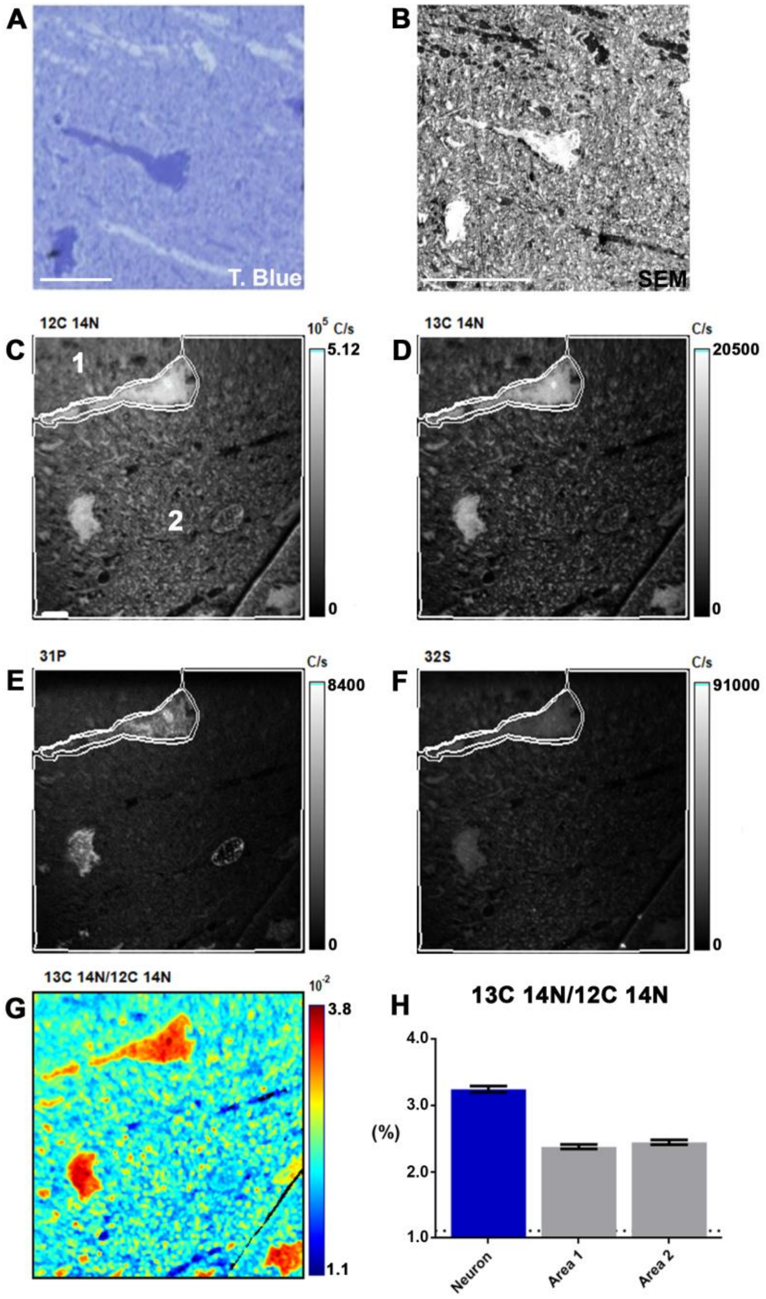

Supplementary Figure 5: ^{13}C enrichment in neuron from APP/PS1 mouse labeled for 10 weeks. (**A**) Optical image of neuron stained with Toluidine Blue (T. Blue) 60X. (**B**) Scanning electron microscope image of the same neuron (2,177X). *Scale bar*, 20 µm in **A-B**. (**C**) $^{12}C^{14}N$ ion map. (**D**) $^{13}C^{14}N$ ion map. (**E**) ^{31}P ion map. (**F**) ^{32}S ion map. (**G**) $^{13}C^{14}N/^{12}C^{14}N$ ion map. ROIs used for quantitation of areas 1 and 2 and the neuron are outlined in white in panels (**C-F**). All SILK-SIMS images (**C-G**) are 60×60 µm. *Scale bar*, 5 µm. (**H**) Normalized values of $^{13}C^{14}N/^{12}C^{14}N$ ratios ± S.D. for ROIs. Normalized values are the raw ratios normalized to the ratio of natural abundance of ^{13}C (1.1) and 0% labeled cells (*Supplemental Material,* **Equation 4**). Standard deviations were calculated as the sum in quadrature of the standard deviation of the average ratios measured for non-labeled material and the Poisson errors of the feature itself (*Supplemental Material,* **Equation 5**). Dashed horizontal line represents natural abundance of ^{13}C.

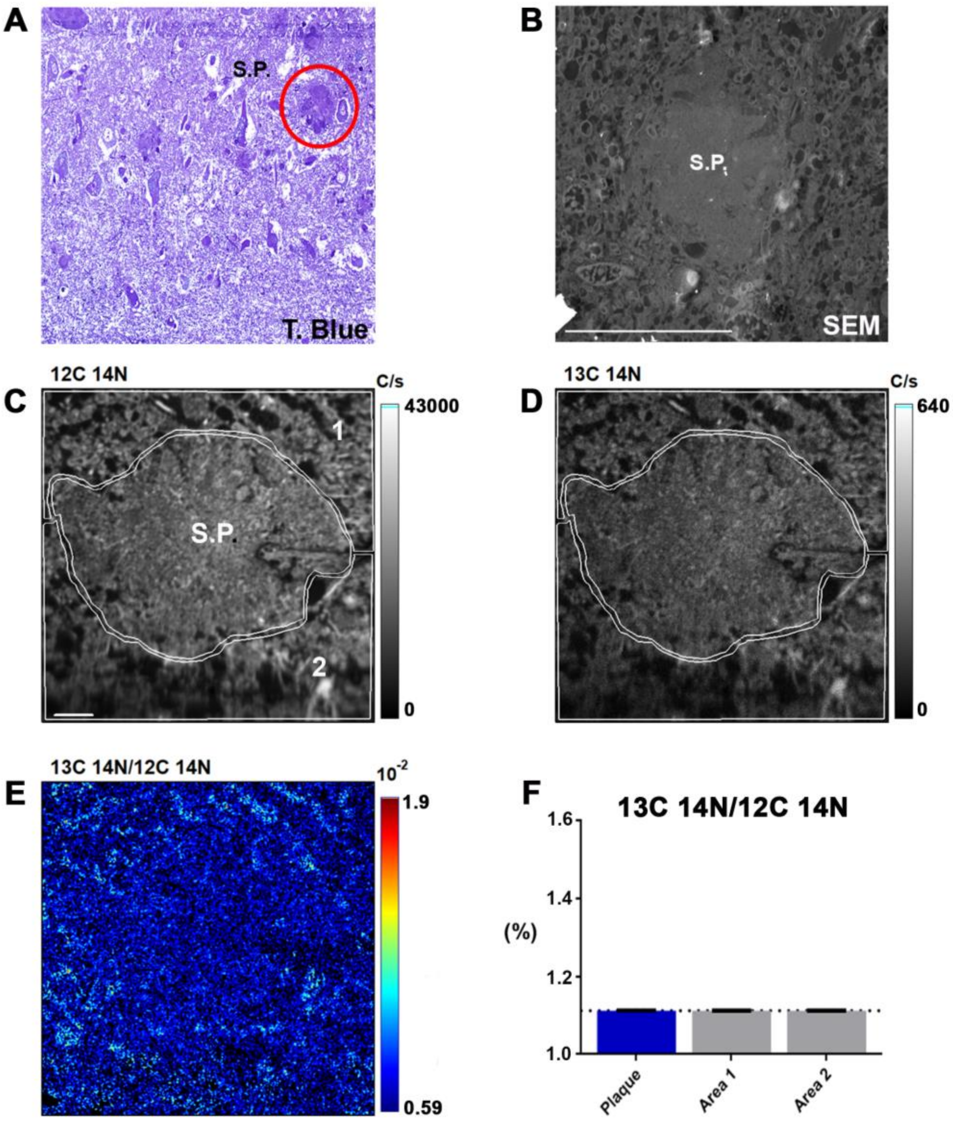

Supplementary Figure 6: Quantitative NanoSIMS imaging of an unlabeled AD participant 1. (A) Optical image of a plaque (S.P., *red*) from Pt1 stained with Toluidine Blue (T. Blue) at 40X. (**B**) Scanning electron microscope image of the same plaque (2,500X). *Scale bar*, 30 µm. (**C**) $^{12}C^{14}N$ ion map. (**D**) $^{13}C^{14}N$ ion map. (**E**) $^{13}C^{14}N/^{12}C^{14}N$ ion map. ROIs used for quantitation of areas 1, 2, and the plaque are outlined in white in panels (**C-D)**. All SILK-SIMS images (**C-E**) are 45×45 µm. *Scale bar*, 5 µm. (**F**) Normalized values of $^{13}C^{14}N/^{12}C^{14}N$ ratios ± S.D. for ROIs. As expected, unlabeled tissue does not show ^{13}C enrichment in the plaque or surrounding areas. Dashed horizontal line represents natural abundance of ^{13}C (1.1%). Ratios were normalized to the ratio of natural abundance ^{13}C and the unlabeled feature itself (*Supplemental Material*, **Equation 4**). The normalized standard deviation represents the sum in quadrature of the standard deviation of the Poisson errors of the feature itself (*Supplemental Material*, **Equation 5**).

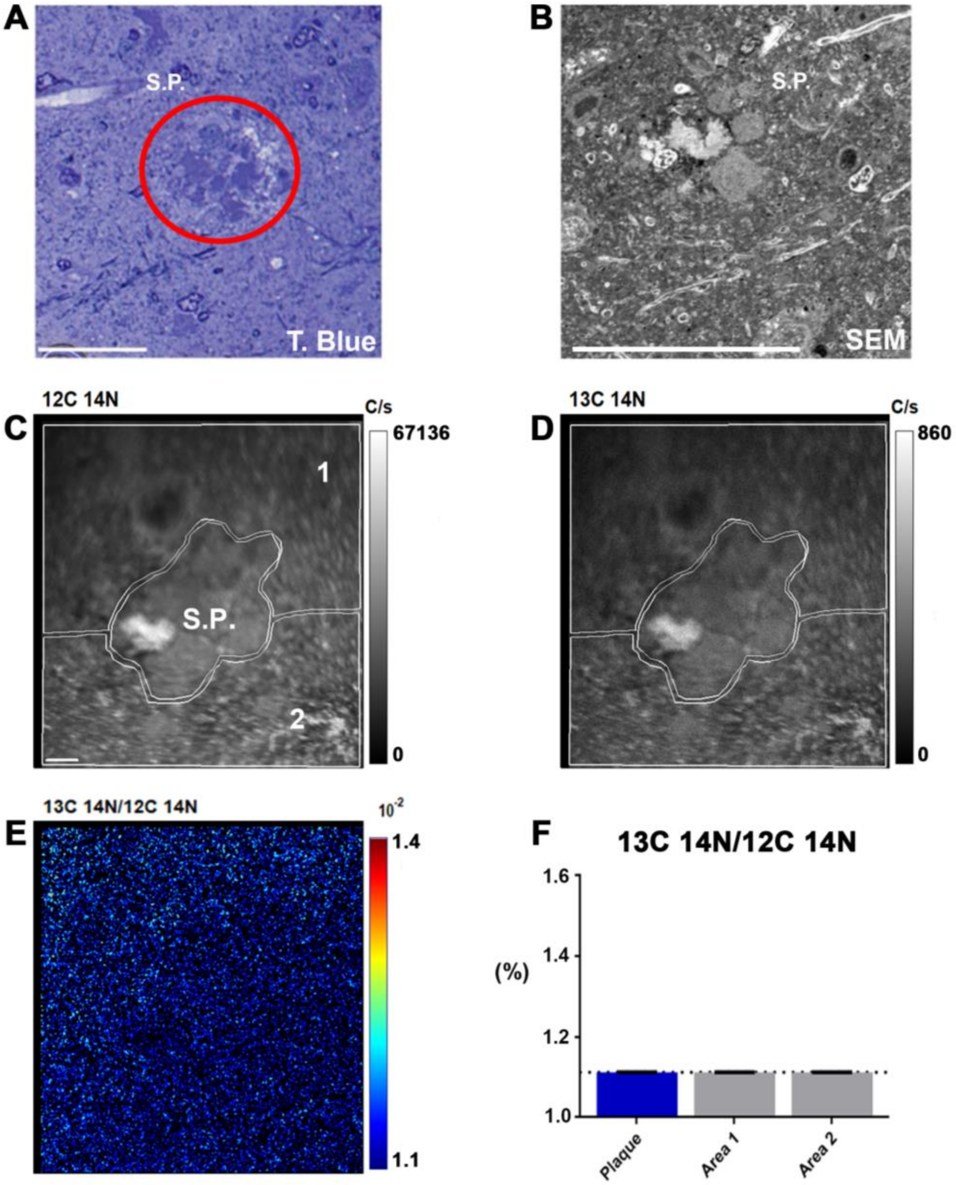

Supplementary Figure 7: Quantitative NanoSIMS imaging of SILK participant 3 with a 4.5 year delta between labeling and expiration. (**A**) Optical image of a plaque (S.P., *red*) from Pt3 stained with Toluidine Blue (T. Blue) at 40X. (**B**) Scanning electron microscope image of the same plaque (1,525X). *Scale bar*, 40 µm in **A**-**B**. (**C**) $^{12}C^{14}N$ ion map. (**D**) $^{13}C^{14}N$ ion map. (**E**) $^{13}C^{14}N/^{12}C^{14}N$ ion map. ROIs used for quantitation of areas 1, 2, and the plaque are outlined in white in panels (**C-D)**. All SILK-SIMS images (**C-E**) are 55×55 µm. *Scale bar*, 5 µm. (**F**) Normalized values of $^{13}C^{14}N/^{12}C^{14}N$ ratios ± S.D. for ROIs. In the areas examined, no ^{13}C enrichment in the plaque or even surrounding areas. If ^{13}C enrichment were present in this area it would be above natural abundance even after normalization. Dashed horizontal line represents natural abundance of ^{13}C (1.1%). Ratios were normalized to the ratio of natural abundance ^{13}C and the unlabeled feature itself (*Supplemental Material*, **Equation 4**). The normalized standard deviation represents the sum in quadrature of the standard deviation of the Poisson errors of the feature itself (*Supplemental Material*, **Equation 5**).

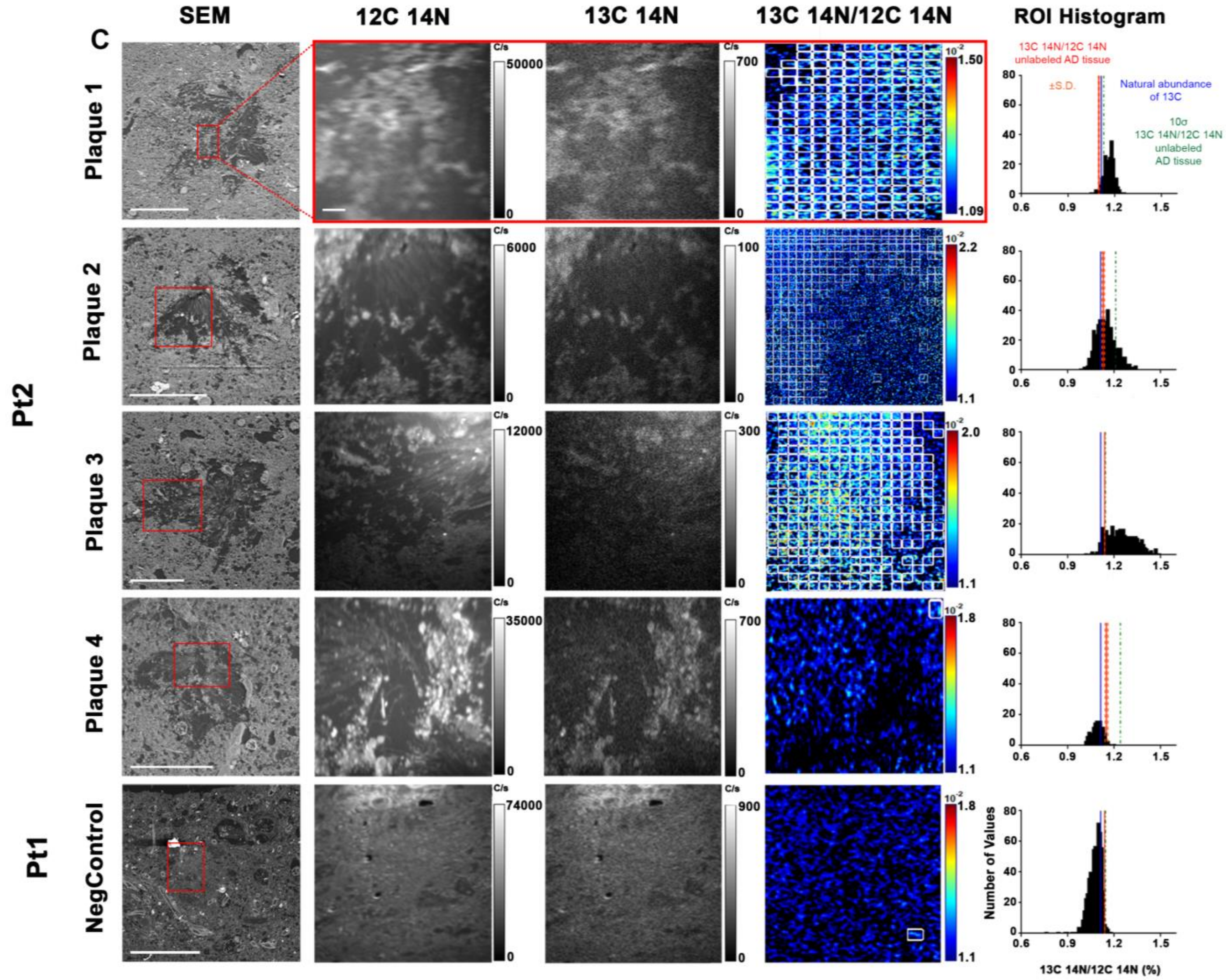

Supplementary Figure 8: ROIs significantly enriched in ^{13}C in participant 2 with an 8 day delta between labeling and expiration. From left to right, scanning electron microscope image (SEM) of the plaque (red box defines area imaged by SILK-SIMS), $^{12}C^{14}N$ ion map, $^{13}C^{14}N$ ion map, $^{13}C^{14}N/^{12}C^{14}N$ ion map, and histogram plot of all ROIs and their respective $^{13}C^{14}N/^{12}C^{14}N$ ratios. The SILK-SIMS image was subdivided into 625 ROIs each representing 10×10 pixels (or 20×20 for plaque 3) from which carbon ratios were calculated. The blue line is the natural abundance of ^{13}C (1.1%), red line the measured $^{13}C^{14}N/^{12}C^{14}N$ ratio of an unlabeled AD brain with ± standard deviation in orange, and the mean of the unlabeled sample+10σ is shown in green. All histogram ratios represent ratios normalized to the ratio of natural abundance ^{13}C and Negative Control (see *Supplemental Material*, **Equation 4**). ROIs that were significantly enriched in ^{13}C as described in the *Methods and Materials* are outlined in white boxes. *Scale bar* for SEM images, 40 μm. *Scale bar* for all SILK-SIMS images, 5 μm. ROIs for each are, Plaque 1: 10×10 pixels (0.95 μm^2); Plaque 2: 20×20 pixels (1.8 μm^2); Plaque 3: 10×10 pixels (3.8 μm^2); Plaque 4: 10×10 pixels (3.0 μm^2); NegControl: 10×10 pixels (3.0 μm^2)

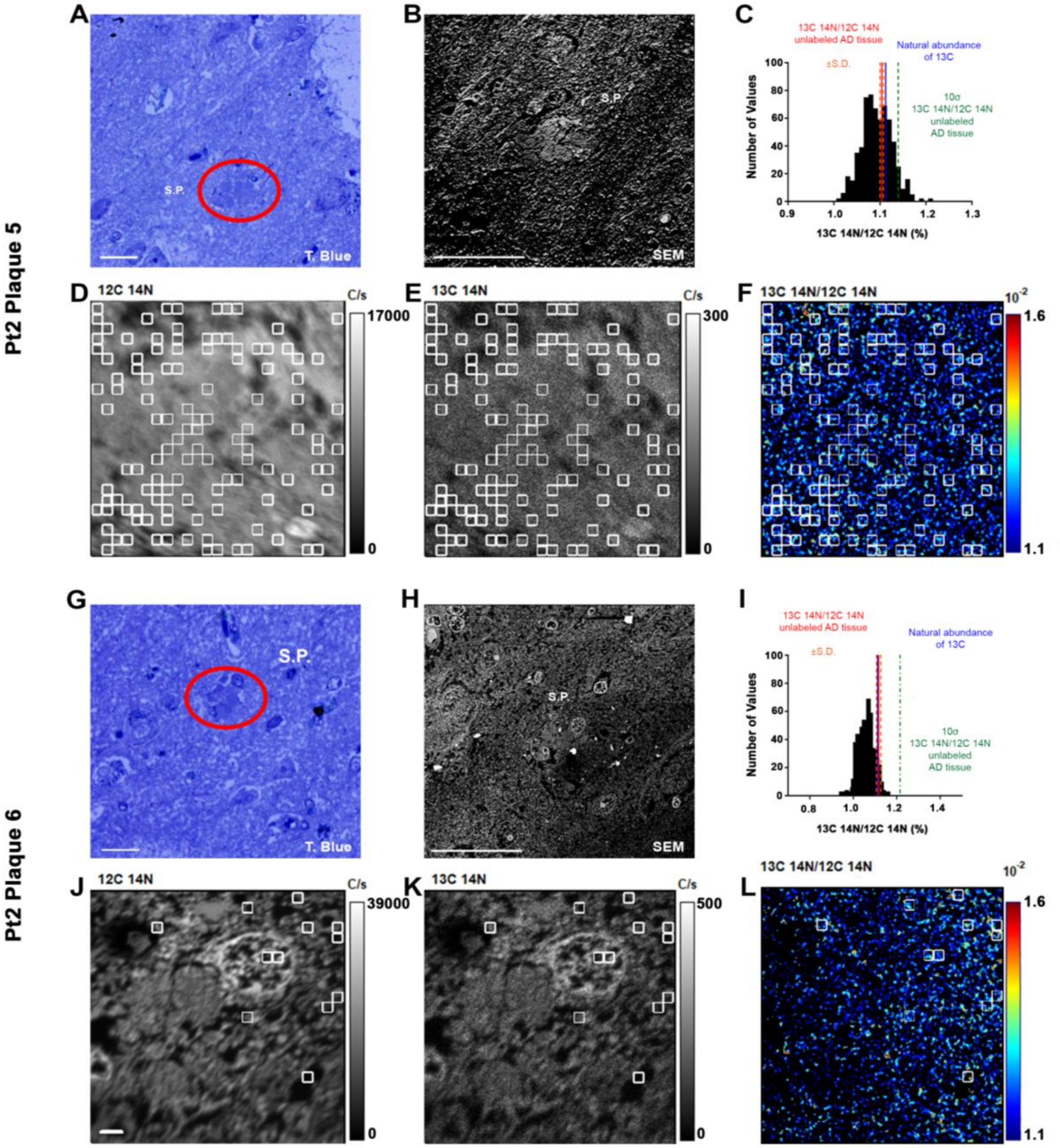

Supplementary Figure 9: Quantitative NanoSIMS imaging of SILK participant 2 plaques in the precuneus. Labeling is apparent in plaques in a separate region of Pt2 brain – the precuneus. The precuneus region is thought to be an area of early deposition in AD (3). (**A**) Optical image of plaque 5 (S.P., *red*) from Pt2 stained with Toluidine Blue (T. Blue) at 40X. (**B**) Scanning electron microscope image of the same plaque (1,248X). (**C**) The SILK-SIMS image was subdivided into 625 ROIs each representing 10x10 pixels from which carbon ratios were calculated. The histogram plots all ROIs and their respective normalized $^{13}C^{14}N/^{12}C^{14}N$ ratios. The blue line is the natural abundance of ^{13}C (1.1%), red line the measured $^{13}C^{14}N/^{12}C^{14}N$ ratio of unlabeled AD brain with ± standard deviation in orange, and the mean of the unlabeled sample+10σ in green. (**D**) $^{12}C^{14}N$ ion map. (**E**) $^{13}C^{14}N$ ion map. (**F**) $^{13}C^{14}N/^{12}C^{14}N$ image showing the distribution of ^{13}C in the sample. ROIs that were significantly enriched in ^{13}C as described in the *Methods and Materials* are outlined in white. *Note*: SILK-SIMS image in panels **D** and **E** are distorted due to a shortage in a lens in the primary column of the instrument at the time of acquisition. This does not affect the quantitation, only the image focus.

(**G**) Optical image of plaque 6 (S.P., *red*) from Pt2 stained with Toluidine Blue (T. Blue) at 40X. (**H**) Scanning electron microscope image of the same plaque (1,250X). *Scale bar*, 40 µm. (**I**) The SILK-SIMS image was subdivided into 625 ROIs each representing 10×10 pixels from which carbon ratios were calculated. The histogram plots all ROIs and their respective $^{13}C^{14}N/^{12}C^{14}N$ ratios. The blue line is the natural abundance of ^{13}C (1.1%), red line the measured $^{13}C^{14}N/^{12}C^{14}N$ ratio of an unlabeled AD brain with ± standard deviation in orange, and the mean of the unlabeled sample+10σ is shown in green. (**J**) $^{12}C^{14}N$ ion map. (**K**) $^{13}C^{14}N$ ion map. (**L**) $^{13}C^{14}N/^{12}C^{14}N$ image showing the distribution of ^{13}C in the sample per pixel. ROIs that were significantly enriched in ^{13}C as described in the *Methods and Materials* are outlined in white. *Scale bar,* 16 µm in **A** and **G**. *Scale bar*, 40 µm in **D** and **H**. All SILK-SIMS images (**D-F and J-L**) are 25x25 µm. SILK-SIMS *Scale bar*, 2 µm. All ROIs represent 10×10 pixels (0.98 µm^2).

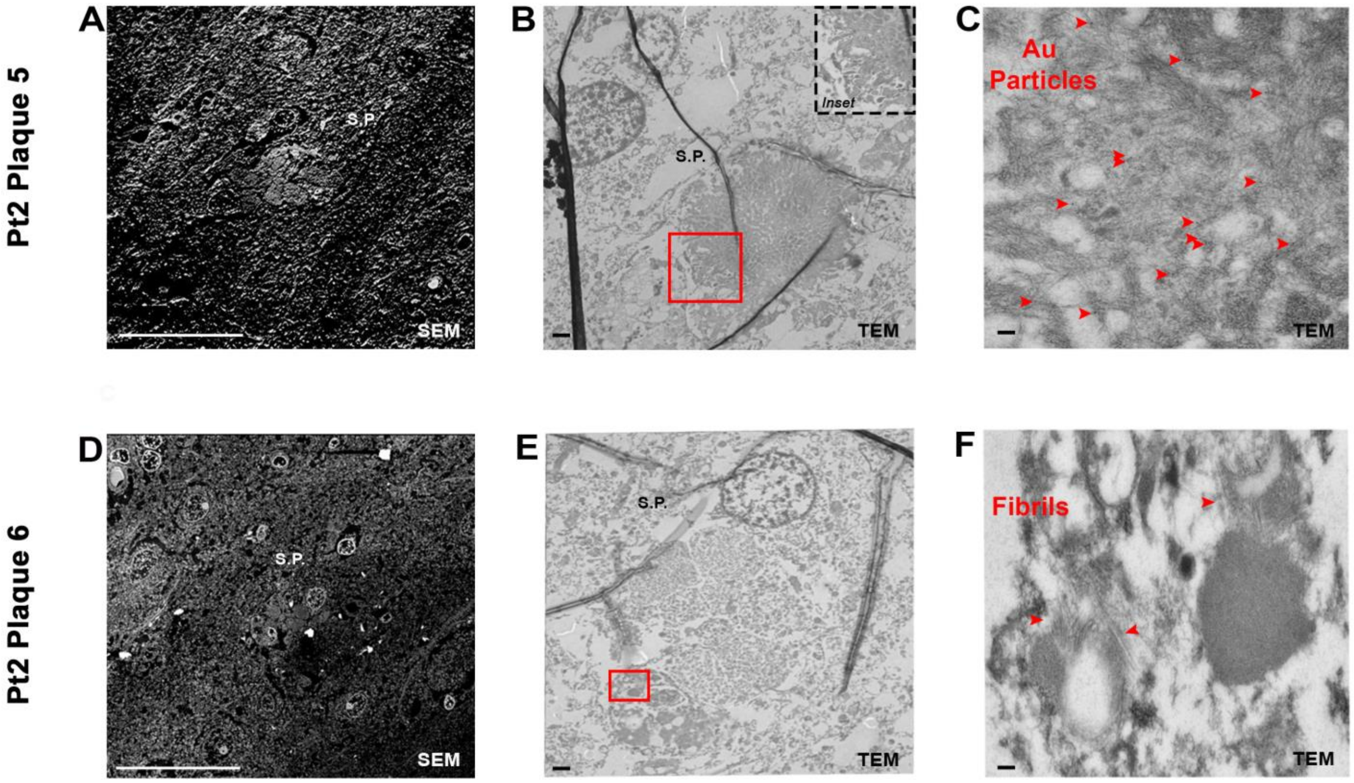

Supplementary Figure 10: Ultra-structure characterization and anti-Aβ immuno-gold labeling of selected plaques in the precuneus. (**A**) Scanning electron microscope image of the Pt2 plaque 5 (1,248X) in the precuneus (*from* **Figure S12B in Supplementary Material**). (**B**) Transmission electron microscope image of the same plaque (1,500X). *Upper right Inset* is a 12,000X magnification of the region outlined in red. (**C**) High magnification (25,000X) of the region outlined in red in panel **B**. Red arrowheads highlight 10 nm gold particles after immuno-labeling with anti-Aβ antibody 82E1 (Aβ epitope 1-16). (**D**) Scanning electron microscope image of the Pt2 plaque 6 (1,250X) in the precuneus (*from* **Figure S12H in Supplementary Material**). (**E**) Transmission electron microscope image of the same plaque (1,500X). (**F**) High magnification (30,000X) of the region outlined in red in panel **e**, red arrowheads highlight fibrils. The Aβ immuno-reactivity seen in plaque 5 and the fibrillar structures highlighted in plaque 6 provide verification that the two features identified in **Figure S12 in Supplementary Material** are indeed amyloid plaques, likely of the dense-core type. *Scale bar*, 40 µm in **A** and **D**, 2 µm in **B** and **E**, and 100 nm in **C** and **F**.

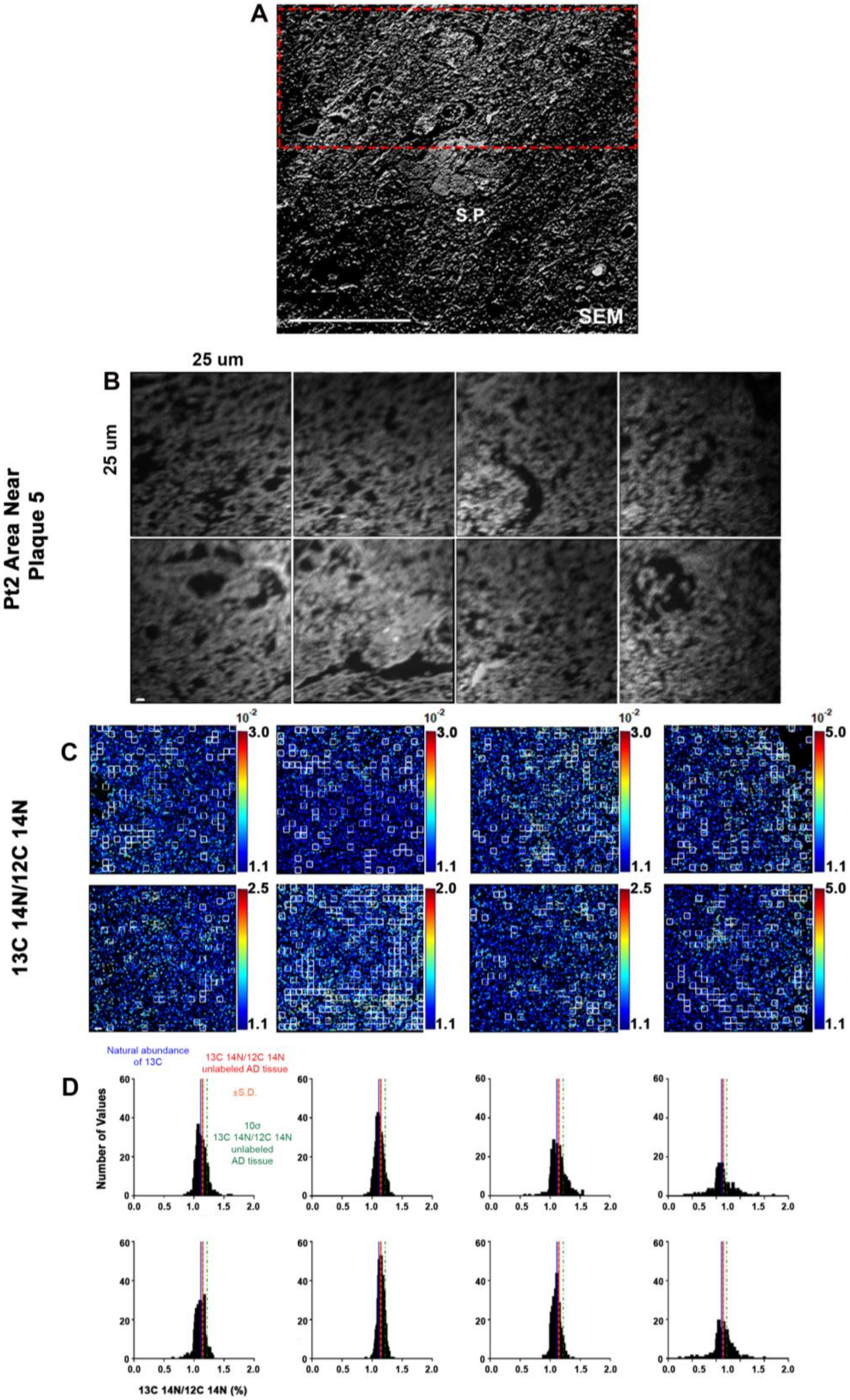

Supplementary Figure 11: Quantitative NanoSIMS imaging of brain parenchyma 50 µm away from plaque 5. (**A**) Scanning electron microscope image (1,248X) of the plaque in **Figure S12A-B in Supplementary Material**. *Scale bar*, 40 µm; red rectangle outlines region near the plaque imaged via SILK-

SIMS. (**B**) Ion map of $^{12}C^{14}N$ in a chained analysis taken near plaque 5 at 25×25 µm. *Scale bar*, 1 µm. (**C**) $^{13}C^{14}N/^{12}C^{14}N$ image showing the distribution of ^{13}C in the sample per pixel. ROIs that were significantly enriched in ^{13}C as described in the *Methods and Materials* are outlined in white. We note higher levels of ^{13}C enrichment more distal to rather than proximal to the plaque. The levels of ^{13}C enrichment were overall lower proximal to the plaque, yet more enriched than the plaque. Evidence of labeling in the brain of Pt2 in areas other than plaques is expected as the stable isotope biomolecule, $^{13}C_6$-leucine, is an essential amino acid and expected to be incorporated other regions. (**D**) Histograms of the ROIs representing 10×10 pixels from each image from which normalized $^{13}C^{14}N/^{12}C^{14}N$ ratio were calculated. The blue line is the natural abundance of ^{13}C (1.1%), red line the measured $^{13}C^{14}N/^{12}C^{14}N$ ratio of unlabeled AD brain with ± standard deviation in orange, and the mean of the unlabeled sample+10σ in green.

2.1 Formulas

The delta (δ) value of a given region-of-interest (ROI) is given in equation 1

$$\delta = \left(\frac{R_{ROI} \ - \ R_{Std}}{R_{Std}} \right) \times 1000 \qquad (1)$$

Where R_{ROI} is the $^{13}C^{14}N/^{12}C^{14}N$ ratio of the ROI in SILK tissue calculated from summing the counts of every pixel contained within the individually defined ROI subtracted from the R_{Std} – the average ratio of repeated measurements on unlabeled tissue. The resulting value is divided by R_{Std} and multiplied by 1000 to yield δ in permil (‰).

By re-arranging equation 1, we derive equation 2

$$R_{ROI} = (\delta / 1000 + 1) \times R_{Std} \qquad (2)$$

which is use to derive the raw $^{13}C^{14}N/^{12}C^{14}N$ ratio for a given ROI (R_{ROI}).

The error (σ_{ROI}) associated with R_{ROI} is derived from the error associated with δ and is given as

$$\sigma_{ROI} = (R_{Std} \times \sigma_{\delta ROI}) / 1000 \qquad (3)$$

where R_{Std} is the average ratio of repeated measurements on unlabeled tissue and $\sigma_{\delta ROI}$ is the δ error (*i.e.*, in permil, ‰) of the ROI of the unknown or labeled sample.

The normalized $^{13}C^{14}N/^{12}C^{14}N$ ratio in a given ROI ($^{n}R_{ROI}$) is given by equation 4

$$^{n}R_{ROI} = R_{ROI} \times \left(\frac{R_{Theo}}{R_{Std}} \right) \qquad (4)$$

Where R_{Theo} is the standard value for carbon-13 based on the natural abundance $^{13}C/^{12}C$ ratio (0.011).

Lastly, the raw error of the ROI is normalized ($^{n}\sigma_{ROI}$) by multiplying the normalized ratio of the sample ($^{n}R_{ROI}$) by the sum in quadrature of the standard deviation of the average ratios measured for non-labeled material, σ_{Std}, and the Poisson errors, σ_{ROI}, of the ROI itself, as given by equation 5

$$^{n}\sigma_{ROI} \ = \ ^{n}R_{ROI} \times \sqrt{\left(\left(\frac{\sigma_{ROI}}{R_{ROI}} \right)^2 + \left(\frac{\sigma_{Std}}{R_{Std}} \right)^2 \right)} \qquad (5)$$

where R_{Std} is the average raw ratio of repeated measurements on unlabeled tissue and R_{ROI} is the raw ratio calculated from summing the counts of every pixel contained within the individually defined ROI.

3.1 Supplementary Datasets

30April2017_Pt2Plaque6_Precuneus_Quant.xlsx
1May2017_Pt2NearPlaque5_Precuneus_Quant.xlsx
5May2017_Pt2Plaque5_Precuneus_Quant.xlsx
21June2017_Pt2Plaque1_Frontal_Quant.xlsx
22June2017_Pt2Plaque2_Frontal_Quant.xlsx
26June2017_Pt1NegControl_Frontal_Quant.xlsx
26June2017_Pt2Plaque3_Frontal_Quant.xlsx
27June2017_Pt2Plaque4_Frontal_Quant.xlsx

4.1 Supplementary References

1. Lechene, C., Hillion, F., Mcmahon, G., Benson, D., Kleinfeld, A.M., Kampf, J.P., Distel, D., Luyten, Y., Bonventre, J., Hentschel, D., Park, K.M., Ito, S., Schwartz, M., Benichou, G., and Slodzian, G. High-resolution quantitative imaging of mammalian and bacterial cells using stable isotope mass spectrometry. *J Biol* (2006) 5: 20. doi:10.1186/jbiol42
2. Alberts, B., Johnson, A., Lewis, J., Raff, M., Roberts, K., and Walter, P. (2002). *Molecular Biology of the Cell.* New York: Garland Science.
3. Braak, H., and Braak, E. Frequency of stages of Alzheimer-related lesions in different age categories. *Neurobiol Aging* (1997) 18: 351-357.
